# Pathological mitophagy disrupts mitochondrial homeostasis in Leber’s hereditary optic neuropathy

**DOI:** 10.1016/j.celrep.2022.111124

**Published:** 2022-07-19

**Authors:** Alberto Danese, Simone Patergnani, Alessandra Maresca, Camille Peron, Andrea Raimondi, Leonardo Caporali, Saverio Marchi, Chiara La Morgia, Valentina Del Dotto, Claudia Zanna, Angelo Iannielli, Alice Segnali, Ivano Di Meo, Andrea Cavaliere, Magdalena Lebiedzinska-Arciszewska, Mariusz R. Wieckowski, Andrea Martinuzzi, Milton N. Moraes-Filho, Solange R. Salomao, Adriana Berezovsky, Rubens Belfort, Christopher Buser, Fred N. Ross-Cisneros, Alfredo A. Sadun, Carlo Tacchetti, Vania Broccoli, Carlotta Giorgi, Valeria Tiranti, Valerio Carelli, Paolo Pinton

**Affiliations:** 1 Department of Medical Sciences, Laboratory for Technologies of Advanced Therapies, University of Ferrara, 44121 Ferrara, Italy; 2 IRCCS Istituto delle Scienze Neurologiche di Bologna, Programma di Neurogenetica, Bologna, Italy; 3 Unit of Medical Genetics and Neurogenetics, Fondazione IRCCS Istituto Neurologico Carlo Besta, Milano, Italy; 4 Centro Imaging Sperimentale, Istituto di Ricovero e Cura a Carattere Scientifico (IRCCS), San Raffaele Scientific Institute, via Olgettina 60, 20132 Milan, Italy; 5 Department of Clinical and Molecular Sciences, Polytechnical University of Marche, Ancona, Italy; 6 Department of Biomedical and Neuromotor Sciences (DIBINEM), University of Bologna, Bologna, Italy; 7 Division of Neuroscience, Istituto di Ricovero e Cura a Carattere Scientifico (IRCCS), San Raffaele Scientific Institute, via Olgettina 60, 20132 Milan, Italy; 8 National Research Council (CNR), Institute of Neuroscience, Milan, Italy; 9 Nencki Institute of Experimental Biology, Polish Academy of Sciences, 3 Pasteur Street, 02-093 Warsaw, Poland; 10 Scientific Institute, IRCCS E. Medea, Department of Conegliano-Pieve di Soligo, Treviso, Italy; 11 Instituto de Olhos de Colatina, Colatina, Espírito Santo, Brazil; 12 Departamento de Oftalmologia e Ciências Visuais, Escola Paulista de Medicina, Universidade Federal de São Paulo (UNIFESP), São Paulo, São Paulo, Brazil; 13 Oak Crest Institute of Science, Monrovia, CA, USA; 14 Doheny Eye Institute, Los Angeles, CA, USA; 15 Department of Ophthalmology, David Geffen School of Medicine at UCLA, Los Angeles, CA, USA; 16 Dipartimento di Medicina Sperimentale, Università degli Studi di Genova, Genoa, Italy

**Keywords:** mitophagy, autophagy, LHON, mitochondria, mtDNA, retinal ganglion cells, optic nerve, cybrids, iPSCs, therapy

## Abstract

Leber’s hereditary optic neuropathy (LHON), a disease associated with a mitochondrial DNA mutation, is characterized by blindness due to degeneration of retinal ganglion cells (RGCs) and their axons, which form the optic nerve. We show that a sustained pathological autophagy and compartment-specific mitophagy activity affects LHON patient-derived cells and cybrids, as well as induced pluripotent-stem-cell-derived neurons. This is variably counterbalanced by compensatory mitobiogenesis. The aberrant quality control disrupts mitochondrial homeostasis as reflected by defective bioenergetics and excessive reactive oxygen species production, a stress phenotype that ultimately challenges cell viability by increasing the rate of apoptosis. We counteract this pathological mechanism by using autophagy regulators (clozapine and chloroquine) and redox modulators (idebenone), as well as genetically activating mitochondrial biogenesis (PGC1-α overexpression). This study substantially advances our understanding of LHON pathophysiology, providing an integrated paradigm for pathogenesis of mitochondrial diseases and druggable targets for therapy.

## Highlights

• Autophagy and mitophagy are abnormally activated in samples carrying LHON mutations
• Autophagy and mitophagy affect LHON cells’ viability
• Therapeutic approaches targeting autophagy reverts LHON cells’ apoptotic death

## Graphical abstract

**Figure:**
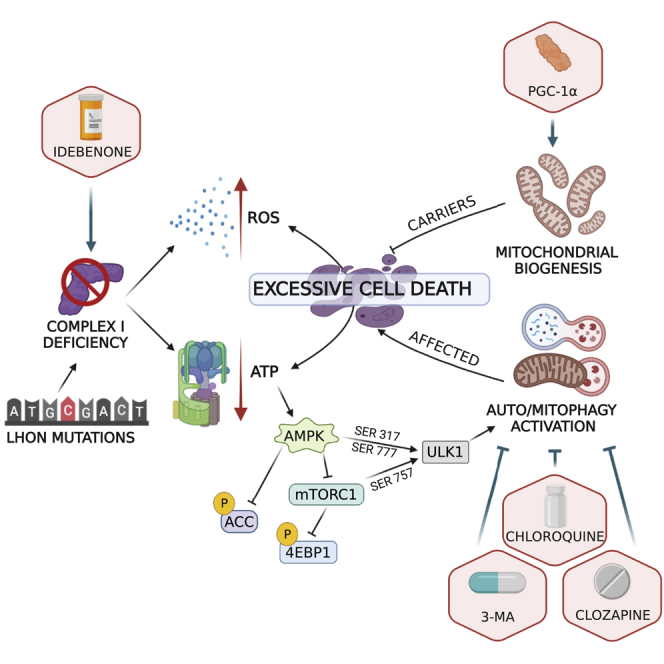

## Teaser

Danese et al. show that autophagy and mitophagy are pathologically increased in Leber’s hereditary optic neuropathy (LHON)-affected individuals. These characteristics reflect a mitochondrial stress phenotype that activates the apoptotic response. Therapeutic strategies balancing the autophagy and mitophagy levels and the mitochondrial homeostasis reverse the pathologic phenotype of LHON-affected cells.

## Introduction

Leber’s hereditary optic neuropathy (LHON) is among the most frequent mitochondrial disorders (Bargiela et al., 2015; Yu-Wai-Man et al., 2016) and, over three decades ago, was the first to be associated with maternally inherited missense mutations affecting mitochondrial DNA (mtDNA) (Wallace et al., 1988). This blinding disease is peculiar, as it very selectively affects only retinal ganglion cells (RGCs), which are the terminal retinal neurons providing the axons to the optic nerve. RGCs undergo a catastrophic wave of neurodegeneration, leading to subacute optic nerve atrophy and severe loss of central vision (Carelli et al., 2004; Yu-Wai-Man et al., 2011). Usually, one of three common LHON mtDNA mutations, all affecting the NADH dehydrogenase (ND) subunits of complex I, are found in the homoplasmic state (100% of mtDNA is mutated) in all individuals from the maternal lineage. However, only a subset of individuals becomes affected (incomplete penetrance), most frequently young males (gender prevalence) (Carelli et al., 2004; Yu-Wai-Man et al., 2011). Complex I dysfunction induced by LHON mutations is reflected in decreased efficiency of oxidative phosphorylation (OXPHOS) (Baracca et al., 2005; Korsten et al., 2010) and increased reactive oxygen species (ROS) production (Beretta et al., 2004; Floreani et al., 2005; Lin et al., 2012). *In vitro*, if cells are forced to rely on OXPHOS for energy production by switching the culture medium carbon source from glucose to galactose, a loss of viability is observed, due to increased apoptosis (Ghelli et al., 2003; Zanna et al., 2005). Furthermore, *ex vivo* (blood cells, muscle biopsies), postmortem (retinal and optic nerve specimens), and *in vitro* (fibroblasts) evidence pointed to increased mitobiogenesis as a compensatory strategy to counteracting the defective phenotype induced by LHON mutations (Giordano et al., 2014).

In LHON, the remarkable tissue specificity has been proposed to depend on the unique characteristic of RGC axons, which are unmyelinated in the long initial stretch of their intra-retinal tract and only then become myelinated as they cross the lamina cribrosa at the optic nerve head (Carelli et al., 2004; Yu-Wai-Man et al., 2011). This myelination pattern implicates an asymmetric energy dependence reflected by the different mitochondrial densities and dynamics needed to correctly distribute the organelles along the RGC axons. Thus, the compensatory increase of mitobiogenesis must be considered in this context. In fact, small RGCs with thin axons are the most sensitive to the defective mitochondrial metabolism present in LHON (Pan et al., 2012; Sadun et al., 2000), and a larger optic disc is a protective factor (Ramos Cdo et al., 2009). The major modifying effect driving the two unexplained features of male prevalence and incomplete penetrance may depend on divergent efficiency in promoting the compensatory mitobiogenesis (Giordano et al., 2014). In particular, estrogens activate mitobiogenesis, protecting females (Giordano et al., 2011), and, aside from gender, the efficient activation of mitobiogenesis that some LHON mutation carriers display predicts the risk for developing the disease, allowing these carriers to be unaffected for their entire lives (Giordano et al., 2014). The degree of mitobiogenesis is most likely determined by specific, still unknown, genetic variants (Carelli et al., 2003) but is also highly modified by interactions with environmental factors, such as tobacco and alcohol exposure (Carelli et al., 2016; Giordano et al., 2015). More recently, some genetic variants have been proposed as modifiers in LHON penetrance, and these involve different pathways occurring only in specific subgroups of families (Jiang et al., 2016; Yu et al., 2020). Overall, the scenario of LHON penetrance remains extremely complex and only partially understood.

New evidence continues to link mitobiogenesis to a wider master program, regulating mitochondrial homeostasis and life cycle (Giorgi et al., 2021; Twig and Shirihai, 2011). The counterpart of mitobiogenesis is mitochondrial clearance, with the elimination of damaged mitochondria by autophagy, i.e., mitophagy (Patergnani and Pinton, 2015). The execution of mitophagy, its role in LHON pathogenesis, and how mitophagy coordinates with compensatory mechanisms remain largely unknown, prompting the current study, which is aimed at elucidating how mitochondrial homeostasis is disturbed by LHON-related complex I dysfunction, thus paving the road for new druggable therapeutic targets. Our results suggest that pathologically increased mitophagy prevails in LHON-affected individuals as opposed to efficient biogenesis, which characterizes the successful compensation in LHON carriers. The latter is driven by the nuclear background, whereas the pathological quality control is directly dependent on the mutant mtDNA, as supported by cybrid experiments, and this general paradigm applies across cell models, from fibroblasts to neuronal cells, and may be targeted by pharmacological interventions.

## Results

### Autophagy is pathologically increased in cells from LHON-affected patients but not in those from unaffected mutation carriers

Our starting point was to investigate how autophagy may be affected by LHON mutations in patient-derived fibroblasts, induced pluripotent stem cell (iPSC)-derived neuronal cell lines, peripheral blood mononuclear cells (PBMCs), and sera samples. To this end, we thoroughly analyzed the two most frequent homoplasmic LHON mutations, the m.3460G>A/*MT-ND1* and the m.11778G>A/*MT-ND4* (herein called, for the sake of brevity, 3460 and 11778 mutations).

**Figure 1:**
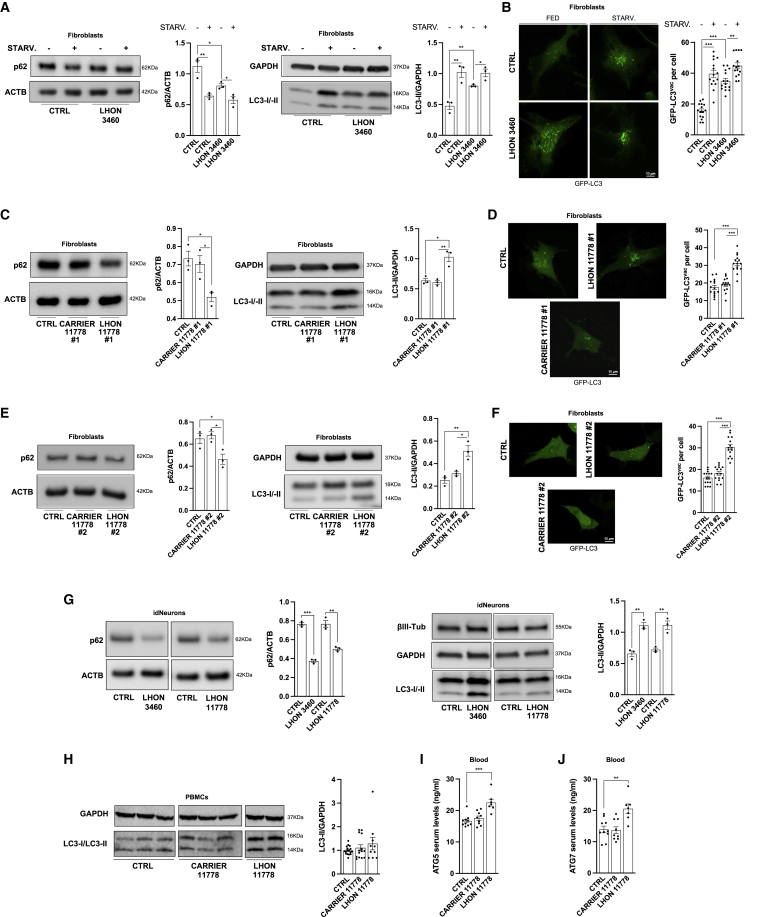
Autophagy is pathologically increased in cells from LHON-affected patients but not in unaffected mutation carriers (A–F) Detection of autophagy activity through immunoblot and GFP-LC3 puncta count in LHON fibroblasts carrying 3460 (A and B) and 11778 mutations (C–F). In the latter, the autophagy levels were also analyzed in fibroblasts obtained from the two unaffected mutation-carrier brothers (identified as #1 and #2). Where not indicated, the representative western blots come from couple #2, while the histograms represent the average of the data from the two pairs #1 and #2. Where indicated, cells were starved (STARV.) for 1 h. (G) Autophagy levels were also detected in idNeurons harboring LHON mutations. (H) Finally, autophagic levels were measured by immunoblot in *ex vivo* PBMCs obtained from healthy individuals (CTRLS) (n = 17), LHON-affected (n = 10), and LHON-carrier (n = 14) (the representative image shown has been cropped to invert the order of samples loading). (I and J) ELISA was performed on serum samples from CTRLS (n = 10), LHON-carrier (n = 9), and LHON-affected (n = 7) patients to detect ATG5 (I) and ATG7 (J). Data are presented as means ± SEM. n = at least 3 independent experiments for western blots or 5 visual fields per at least 3 independent samples per condition for GFP-LC3 experiments. ^∗^p < 0.05, ^∗∗^p < 0.01, and ^∗∗∗^p < 0.001.

Light chain 3 (MAP1LC3, hereafter referred to as LC3) is a specific marker for monitoring autophagy. During autophagy, the cytoplasmic form of this protein (LC3-I) is cleaved and lipidated into the membrane-bound form (LC3-II), which is localized to the autophagosome (Thukral et al., 2015) and correlates with the number of autophagosomes. As an additional autophagy marker, we used the protein SQSTM1/p62, which is inversely correlated with autophagy activity (Katsuragi et al., 2015). We found that in basal conditions, compared with control cells, fibroblasts from an LHON-affected patient (herein abbreviated as LHON-affected) with the 3460 mutation, considered the most severe for biochemical dysfunction (Carelli et al., 1997), presented a sustained autophagy activation (Figure 1A). To further validate these results, we used nutrient-starvation conditions, known to promote the activation of the autophagy machinery (Chen et al., 2014). Under these conditions, autophagy was significantly activated in control fibroblasts, whereas the magnitude of this response was attenuated in LHON-affected fibroblasts, suggesting that, from their high basal autophagy activity, the capacity to further increase did not exceed the top level of controls (Figure 1A). Fluorescence microscopy measurements of autophagy with the GFP-LC3 probe, in basal conditions and after starvation, confirmed these findings (Figure 1B). Autophagy levels in control fibroblasts (n = 4) used for this study were comparable (Figures S1A and S1B), confirming that the differences observed with LHON-affected were dependent on the mtDNA LHON mutations.

To confirm that increased autophagy is a key feature of patients affected by LHON, we also evaluated fibroblasts from two LHON-affected and the respective two brothers, unaffected mutation carriers (herein abbreviated as LHON-carrier), all harboring the most frequent but biochemically milder 11778 mutation (Carelli et al., 1997). While significantly reduced p62 level and increased LC3-II amount were confirmed in the LHON-affected (Figures 1C–1F, respectively, for the first and second pairs of LHON-affected/carrier siblings), as seen for the 3460 mutation, the LHON-carriers had an autophagy activity similar to controls, despite the presence of the homoplasmic 11778 mutation.

As LHON is primarily characterized by the subacute degeneration of RGCs, we sought to test a neuronal cell model. To this aim, we generated different transgene-free iPSC clones by using Sendai-virus-mediated expression of the four Yamanaka’s factors (OCT3/4, SOX2, c-MYC, and KLF4) to reprogram the fibroblasts of two patients carrying the 3460 and the 11778 LHON mutations, respectively, and two controls. Then, we obtained neural precursor cells (NPCs) through embryoid body formation and generated terminally differentiated neurons (Figure S1C). We assessed autophagy activity in 35-day-old iPSC-derived neurons (herein abbreviated as idNeurons) expressing specific markers such as βIII-tubulin, Map2, and NeuN (Figure S1D), and again we observed a significant reduction of p62 and a parallel increase of LC3-II in both LHON-affected carrying the 3460 and the 11778 mutations (Figure 1G).

Autophagosome accumulation, and thus increased levels of LC3, may also occur due to impaired autophagosome degradation (Mizushima et al., 2010). To assess this alternative scenario, we performed autophagy flux analysis in the presence of bafilomycin A1 (Baf-A1) (100 nM for 2 h), which inhibits the late phase of autophagy (Klionsky et al., 2021). We found that Baf-A1 treatment induced abundant cleaved LC3 accumulation in all the fibroblasts analyzed (Figures S1E for 3460 mutation and S1F for 11778 mutation), indicating that the autophagy response is unaffected in our experimental conditions.

To verify if pathological activation of autophagy also characterizes LHON patients, we evaluated *ex vivo* LC3-II amounts in PBMCs derived from controls, LHON-affected, and LHON-carrier homoplasmic for the 11778 mutation (Figure 1H). Due to a widespread variability and a limited number of samples, we failed to observe a significant increase of LC3-II in LHON-affected compared with controls and LHON-carriers (Figure 1H). The ELISA detection of autophagy markers in blood samples represents a more sensible and reliable method to assess autophagy activity in biological samples (Patergnani et al., 2018, 2021a; Xue et al., 2020). Thus, we also evaluated serum levels of the autophagy markers ATG5 and ATG7, highlighting their significant increase only in LHON-affected (Figures 1I and 1J).

Taken together, all these results demonstrated that the autophagy machinery is intrinsically activated in LHON-affected, whereas a compensatory mechanism must be active in LHON-carrier cells, which behaved more similarly to controls.

### Selective autophagy of mitochondria (mitophagy) follows the same pattern of autophagy

**Figure 2:**
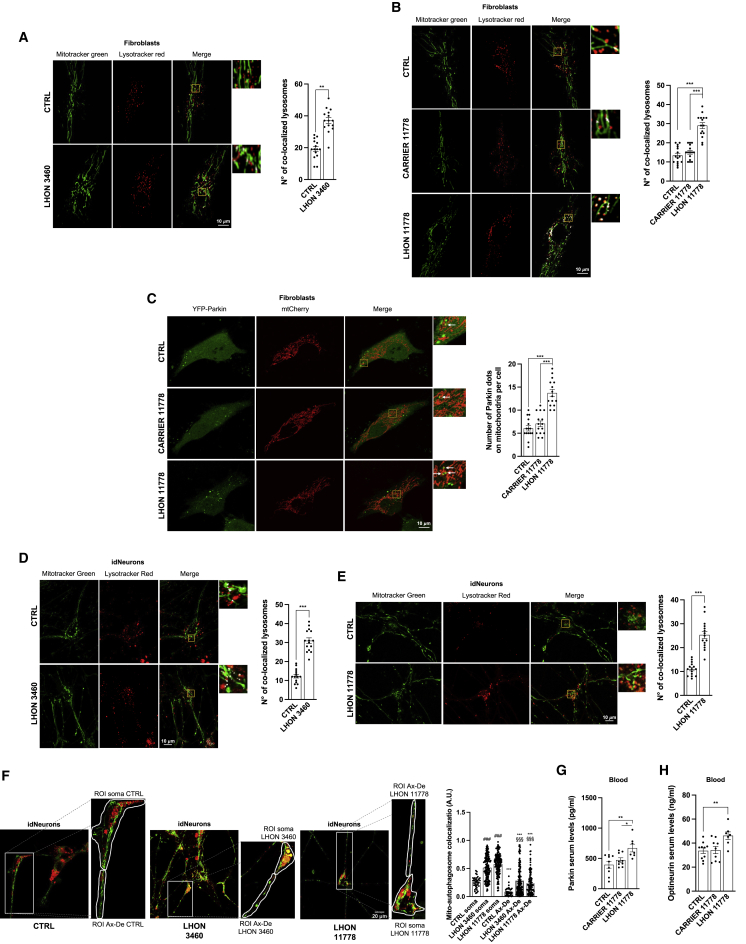
LHON disease is characterized by excessive mitophagy levels (A, B, D, and E) Confocal microscopy assessment of mitophagy respectively in fibroblasts and 35-day-old idNeurons carrying 3460 (A and D) and 11778 (B and E) mutations were performed by loading cells with LysoTracker red and MitoTracker green to visualize lysosomes and mitochondria, respectively. (C) Mitophagy levels were analyzed by detecting the amount of fluorescent YFP-Parkin localized on the mitochondrial surface in fibroblasts from one LHON-affected patient carrying the 11778 mutation and of the non-affected (carrier) brother carrying the same 11778 mutation. (F) A similar methodological approach with LysoTracker red and MitoTracker green was used to investigate whether mito-autophagosomes were present in specific regions of the idNeurons (soma and axon-dendrite (Ax-De) regions). ###p < 0.001 to CTRL soma, §§§p < 0.001 to CTRL Ax-De, ^∗∗∗^p < 0.001 Ax-De to its own soma. (G and H) Increased levels of mitophagy marker Parkin (G) and Optineurin (H) were detected in serum samples of 11778 LHON-affected patients (n = 7) compared with CTRLS (n = 10) and 11778 LHON-carriers (n = 9). Data are presented as means ± SEM. n = at least 5 visual fields per at least 3 independent samples per condition for colocalization experiments. Data to evaluate region-specific mitophagy were obtained from 78 regions of interest (ROIs) for iPSC-derived control neurons, 297 ROIs for iPSC-derived 3460 neurons, and 225 ROIs for iPSC-derived 11778 neurons and analyzed using one-way ANOVA. ^∗^p < 0.05, ^∗∗^p < 0.01, and ^∗∗∗^p < 0.001.

Autophagy also exists in selective forms, which, in the case of mitochondria, removes damaged organelles through the targeted process of mitophagy. We assessed mitophagy by the simultaneous labeling of mitochondria with MitoTracker green and of autophagolysosomes by LysoTracker red (Kanki and Okamoto, 2014; Patergnani and Pinton, 2015). As shown in Figures 2A and 2B, mitophagy was significantly increased in LHON-affected fibroblasts with both 3460 and 11778 mutations, whereas it was not activated in the LHON-carriers with 11778 mutation, displaying an activity similar to control cells. Upon acute mitochondrial dysfunction, the PINK1-Parkin pathway is activated by Parkin recruitment from the cytosol to the mitochondrial surface, ultimately leading to mitophagy (Narendra et al., 2010; Vives-Bauza et al., 2010). By using fluorescent microscopy techniques, we investigated Parkin recruitment to mitochondria, finding that LHON fibroblasts displayed a higher co-localization rate between Parkin and mitochondria than both control and LHON-carrier samples (Figure 2C). The assessment of mitophagy in idNeurons carrying either 3460 or 11778 mutations paralleled the fibroblasts results, displaying a significant increase in LHON-affected compared with controls (Figures 2D and 2E). Recent investigations suggested that during neurodegeneration, the excessive mitochondrial removal characterizing the neuronal cells might be region specific (Zaninello et al., 2020). We investigated this aspect by comparing the mitophagy activity measured in the body and in the axonal-dendrites regions in our massive neuronal culture. This specific growth condition prevented us from clearly identifying the axonal hillock region. Nevertheless, our analysis unveiled elevated mitophagy levels in the soma of the neurons, which also includes the axonal hillock. Although this region-specific mitophagy was a common feature shared between control and LHON-affected neurons (Figure 2F), the latter showed an extremely elevated mitophagy removal of mitochondria compared with control neurons.

Further support to the increased mitophagy activity is provided by the *ex vivo* assessment of Parkin and Optineurin in serum from circulating blood of LHON-affected patients (Figures 2G and 2H).

We attempted to conduct a transmission electron microscopy (TEM) assessment of RGCs in *postmortem* retinas from LHON-affected carrying either the 3460 or 11778 mutation; these tissues are not only difficult to collect, but they are processed several hours after death, thereby making it difficult to have a sufficient number for reliable quantification of the cellular events observed as well as a high resolution and quality of the samples imaged. However, acquisitions with qualitative images are available at Mendeley Data: https://doi.org/10.17632/83vvm47z2f.1.

Overall, this set of experiments documented that the increase of autophagy activity is also reflected on mitophagy, which was enhanced in LHON-affected in cultured cell models, including idNeurons. Again, the LHON-carrier did not display this phenotype, behaving much more closely to controls.

### Pathological autophagy and mitophagy is transferred to cybrids with the LHON mutant mtDNA independently from the affected/carrier status

To establish whether the pathological increase in autophagy and mitophagy observed in the previous experiments is primarily driven by the mutant mtDNA, we evaluated these readouts in a cytoplasmic *trans*-mitochondrial hybrid (cybrid) cell model, a valuable model for investigating mtDNA-dependent phenotypes, removing the influence of the original nuclear background of the patient (King and Attardi, 1989; King et al., 1992; Vergani et al., 1995).

We assessed autophagy in LHON cybrids carrying either the 3460 or 11778 mutation but also the milder m.14484T>C/*MT-ND6* (herein 14484) mutation, generated from LHON-affected fibroblasts compared with mtDNA haplogroup-matched control cybrids. Furthermore, the same experiments were also performed in cybrids generated from a pair of discordant brothers (from #2 independent fibroblast cell lines) carrying the same 11778 homoplasmic mutation, compared with mtDNA haplogroup-matched control cybrids.

**Figure 3:**
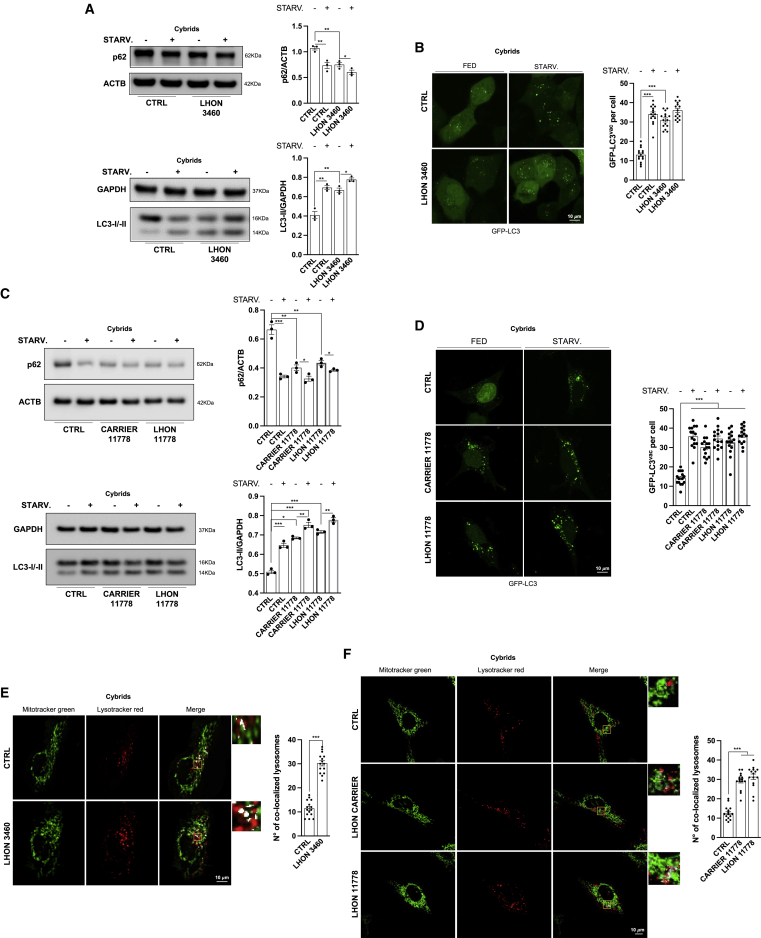
Autophagy and mitophagy results increased in cybrids (A–D) Autophagy detection by immunoblot (A) and fluorescent microscopy (B) was performed in cybrids carrying 3460 and in cybrids derived from fibroblasts from one LHON-affected patient carrying the 11778 mutation and of the non-affected (carrier) brother carrying the same 11778 mutation (C and D). Where indicated, the cells were STARV. for 1 h. (E) Confocal microscopy assessment of mitophagy in control and mutant cybrids harboring 3460 LHON mutations. (F) Similar experiments were achieved in LHON-affected cybrids carrying the 11778 mutation and of the non-affected (carrier) brother carrying the same 11778 mutation. Data are presented as means ± SEM. n = at least 3 independent experiments for western blots or 5 visual fields per at least 3 independent samples per condition for fluorescent microscopy experiments. ^∗^p < 0.05, ^∗∗^p < 0.01, and ^∗∗∗^p < 0.001.

In LHON cybrids, we confirmed the results previously obtained in LHON-affected fibroblasts. LHON mutations significantly increased autophagy activity (Figures 3A–3D, S2A, and S2B), and similar results were also obtained with cybrids carrying the mildest 14484 mutation (Figures S2C and S2D). In addition, the assessment of mitophagy activity in LHON cybrids carrying the 3460 or 11778 mutation also showed significantly increased mitophagy (Figures 3E, 3F, and S2E). Differently, the results obtained in LHON-carrier cybrids were discordant from those previously achieved in fibroblasts. Indeed, LHON-carrier cybrids had both autophagy (Figures 3C and 3D) and mitophagy (Figures 3F and S2E) comparable to the LHON-affected cybrids.

Autophagy flux analysis, also performed by fluorescence microscopy with the tandem mCherry-GFP-LC3 construct, confirmed again that this process was unaffected and that autophagosomes were properly degraded (Figures S3A–S3C).

To further dissect the molecular mechanisms leading to altered autophagy in LHON cells, in particular all potential factors that impinge upon global mitochondrial homeostasis and that co-regulate mitophagy, we investigated the pathways involved in the mechanistic target of rapamycin (mTOR) kinase complex and 5′ adenosine monophosphate-activated protein kinase (AMPK) axis. The mTOR pathway, through phosphorylation events, strongly suppresses autophagy (Missiroli et al., 2016; Xue et al., 2020). Thus, we evaluated whether LHON mutations alter the mTOR/AMPK pathway by assessing AMPK phosphorylation levels and the regulation of mTOR-downstream targets, including the phosphorylation/inactivation of the mRNA translation repressor 4E-binding protein (4EBP1) and the autophagy regulator Unc-51 like autophagy activating kinase (ULK1), a serine/threonine (Ser/Thr) kinase that plays a specific role in clearing mitochondria (Egan et al., 2011). mTOR and AMPK-dependent ULK1 phosphorylation regulates the activity of this pathway (Kim et al., 2011) and the accumulation of dysfunctional mitochondria upon mitophagy induction (Wu et al., 2014) (Figure S3D). As shown in Figure S3E, in LHON cells, sustained autophagy involved AMPK activation and concomitant mTOR pathway inhibition, as evidenced by increased AMPK-mediated phosphorylation of ULK1 in Ser 317 and of acetyl-coenzyme A (CoA) carboxylase (ACC) and decreased phosphorylation of acetyl-eukaryotic translation initiation factor 4EBP1.

In summary, the phenotype of dysfunctional autophagy and mitophagy is tightly associated with the LHON mutations, as it was faithfully transferred with mutant mtDNA in the constant nuclear background of the cybrid cell model and separated from the influence of the original nuclear background of the patient. This phenotype was correlated with the modulation of the mTOR/AMPK pathway.

### Pathological autophagy and mitophagy in LHON reflects a mitochondrial stress phenotype

**Figure 4:**
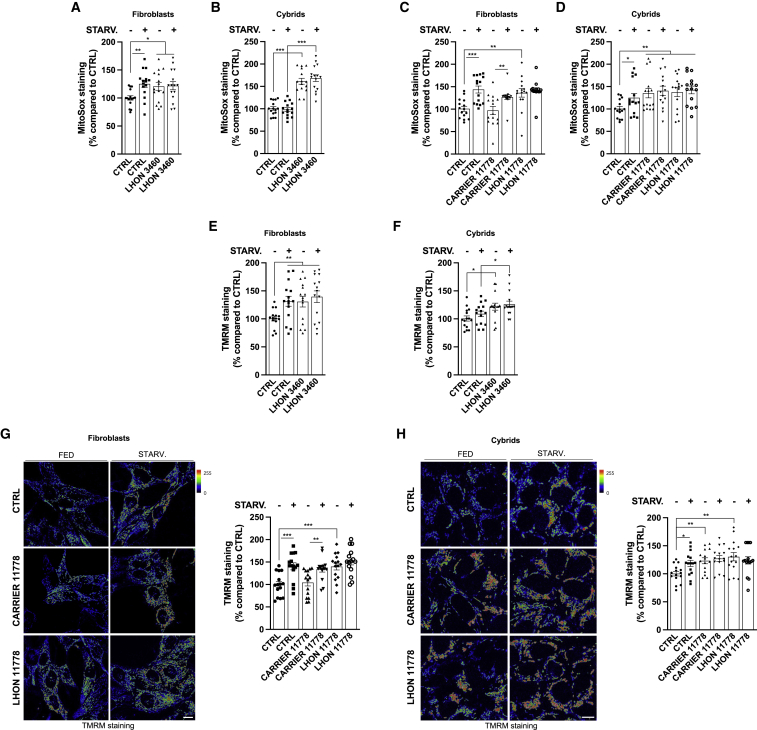
Complex I deficiency leads to altered mitochondrial function in LHON-affected individuals, which is compensated in carriers (A–D) Measurements of mitochondrial ROS production in LHON-derived 3460 fibroblasts (A) and cybrids (B) and in 11778 fibroblasts (C) and cybrids (D) by using MitoSOX red probe. (E–H) The mitochondrial transmembrane potential (Ψ_m_) of 3460 and 11778 fibroblasts and cybrids was detected by using the Ψ_m_-sensitive probe TMRM. When indicated, the cells were STARV. for 30 min before TMRM loading. Data are presented as means ± SEM. n = at least 5 visual fields per at least 3 independent samples per condition. ^∗^p < 0.05, ^∗∗^p < 0.01, and ^∗∗∗^p < 0.001.

Complex I dysfunction is commonly associated with increased ROS production, specifically superoxide (Fiedorczuk and Sazanov, 2018; Hirst and Roessler, 2016). We assessed ROS by using MitoSOX Red, a fluorogenic dye specifically targeted to mitochondria in live cells. All LHON-affected cells (fibroblasts and cybrids) carrying either the 3460 (Figures 4A and 4B) or the 11778 (Figures 4C and 4D) mutation presented increased ROS levels, and a state of nutrient deprivation increased ROS (Figures 4A–4D), corroborating that starvation-induced autophagy is regulated by ROS (Li et al., 2013). Moreover, while ROS production in the LHON-carrier fibroblasts was equivalent to control cells (Figure 4C), excessive ROS levels characterized LHON-carrier cybrids, comparable to LHON-affected cells (Figure 4D).

Notably, a harmful ROS production may be highly dependent on the electrochemical gradient that forms across the inner mitochondrial membrane and sustains mitochondrial membrane potential (Ψ_m_) (Giorgi et al., 2018b; Suski et al., 2012). As shown in Figures 4E and 4F, we found that in 3460 LHON-affected fibroblasts and cybrids, Ψ_m_ appeared significantly increased. Moreover, starvation increased the Ψ_m_ in controls to levels comparable to LHON cells at resting conditions, whereas it failed to affect LHON fibroblasts, which remained hyperpolarized, similar to resting conditions (Figures 4E and 4F). Comparable findings were observed in fibroblasts and cybrids carrying the 11778 mutation (Figures 4G and 4H). Consistent with the ROS results, the LHON-carrier fibroblasts had Ψ_m_ equivalent to controls (Figure 4G), while LHON-carrier cybrids displayed values comparable to the LHON-affected cybrids (Figure 4H).

Overall, these results demonstrate that mitochondria present a stress phenotype in LHON-affected cells characterized by increased ROS and hyperpolarization of Ψ_m_. These features were conserved in LHON-carrier cybrids but were minimal or absent in LHON-carrier fibroblasts, which displayed fairly normal ROS levels and Ψ_m_ similar to controls (Figures 4C and 4G), indicating again an efficient compensatory response to the LHON mutations.

### Therapeutic strategies reverse the pathologic phenotype of LHON-affected cells, balancing mitochondrial homeostasis

LHON pathogenic mutations ultimately cause impaired cell viability and increased rate of mitochondria-dependent apoptosis in cybrids, particularly when cells are pushed into a stress condition, such as in galactose medium (Ghelli et al., 2003; Zanna et al., 2005). Besides cybrids, increased propensity to apoptosis has been reported to occur also in iPSC-derived RGCs carrying an unusual combination of LHON mutations (Wong et al., 2017).

We confirmed here that LHON mutations reduce the growth rate of LHON-affected fibroblasts compared with controls (Figures S4A and S4B), and this effect was transferred in cybrids (Figures S4C and S4D). This was paralleled by a significant increase in apoptosis of both fibroblasts and cybrids, as evidenced by the increased cleavage of poly (ADP-ribose) polymerase 1 (PARP) and caspase 3 (CASP3) (Figures S4E and S4F). The increased propensity to apoptosis also characterized the idNeurons of LHON-affected carrying the 11778 and 3460 mutations, compared with controls (Figure S4G).

Growing evidence points to an intimate relationship linking apoptosis and autophagy (Bialik et al., 2018), suggesting that the activation of autophagy and mitophagy pathways observed in LHON mutant cells may, in turn, also modulate the apoptotic machinery. Thus, we hypothesized that by modulating the autophagy and mitophagy pathways to limit their excessive activation, we could impinge therapeutically on the LHON pathogenic mechanism, correcting the apoptotic loss of cell viability. Autophagy can be finely adjusted with pharmacological interventions targeting either the early or late stages of this process (Choi et al., 2013). The most used inhibitor targeting the early stages is 3-methyladenine (3-MA); for later stages, chloroquine (CQ) and its derivatives are the most commonly used. Recently, a novel class of drugs has been added, i.e., antipsychotic and antidepressant drugs such as clozapine (CL), which acts as potent late-stage autophagy inhibitor (Park et al., 2012; Patergnani et al., 2021b).

**Figure 5:**
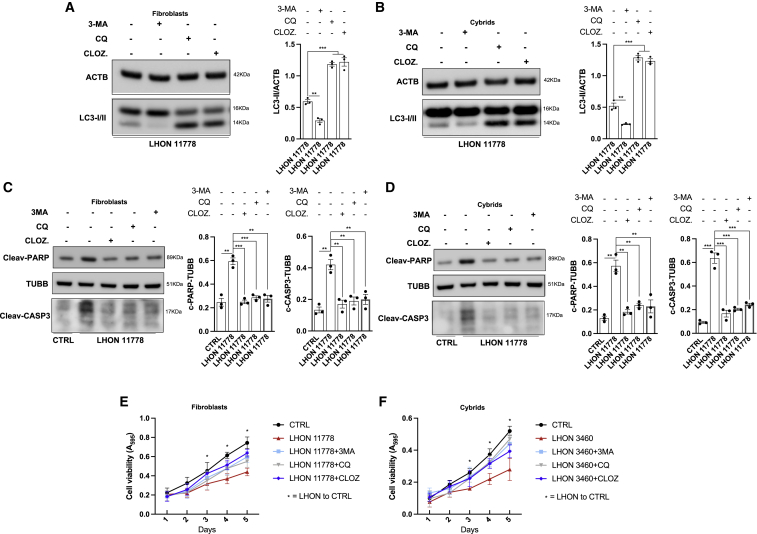
Compensatory therapeutic approaches targeting autophagy reverts LHON cells’ predisposition to apoptotic death (A and B) 11778 fibroblasts (A) and cybrids (B) harboring LHON mutations were treated with different autophagic inhibitors (3-MA [inhibitor of autophagy at early steps], chloroquine [CQ], and clozapine [CLOZ] [inhibitors of autophagy at late steps]). After 48 h, detection of autophagic activity through immunoblot technique was performed. (C and D) Detection of apoptotic process activity on 11778 LHON fibroblasts (C) and cybrids (D) treated with anti-autophagic agents by immunoblotting with antibodies against PARP and CAS3 apoptotic markers. (E and F) Cell viability in fibroblasts (E) and cybrids (F) pretreated with anti-autophagy compounds was performed at different time points. Data are presented as means ± SEM. n = at least 3 independent experiments. ^∗^p < 0.05, ^∗∗^p < 0.01, and ^∗∗∗^p < 0.001.

We tested the efficacy of these drugs for their capacity to interfere with the autophagy machinery, aware of possible re-purposing for some of these molecules. The autophagy inhibitor treatments significantly reduced the autophagy activity, assessed as LC3-II content, in both LHON-affected fibroblasts and cybrids carrying the 11778 mutation (Figures 5A and 5B). Indeed, the early-stage autophagy inhibitor 3-MA significantly decreased the LC3-II levels, whereas CQ and CL, which interfere with lysosome/autophagosome fusion, led to accumulation of LC3-II. Under these treatment conditions, we also found that the extent of apoptosis induction was significantly limited, as evidenced by reduced levels of both cleaved PARP and cleaved CASP3 (Figures 5C and 5D), ultimately increasing cell viability of fibroblasts and cybrids (Figures 5E and 5F). We did not observe any cytotoxic effect of the compounds tested in either control fibroblasts or cybrids (Figure S4H).

**Figure 6:**
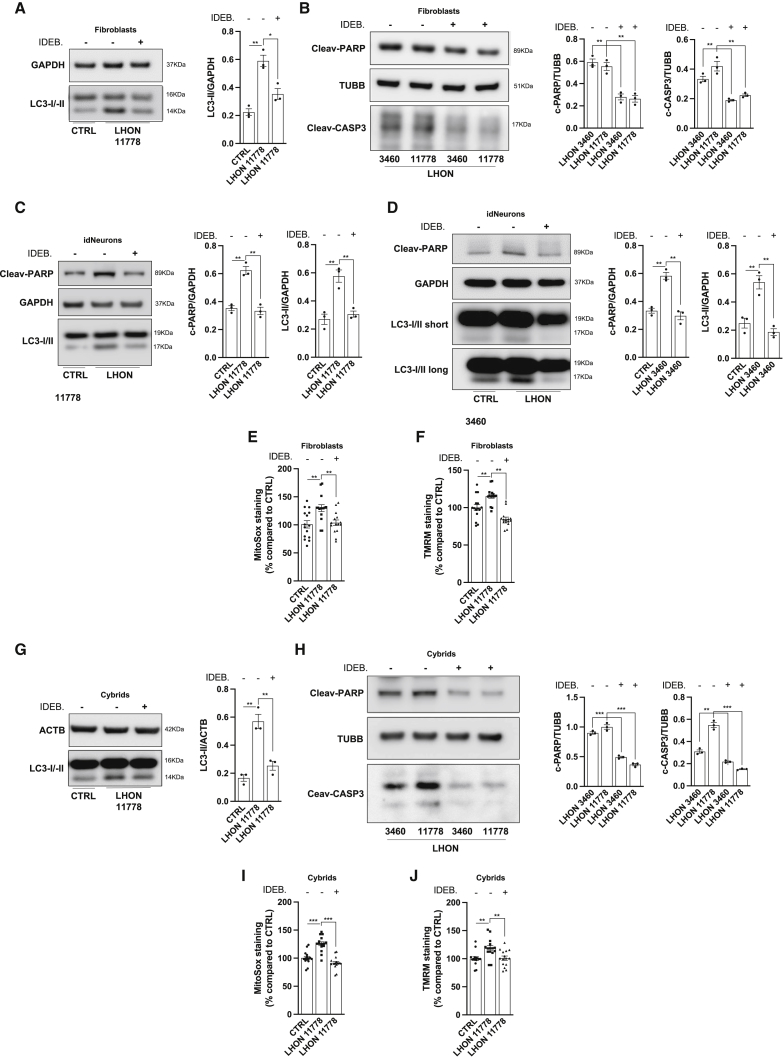
Oxidative stress modulation contributes to decrease in LHON cells’ autophagic activity and apoptotic death (A–D) After treatment with reduced idebenone (IDEB.) (10 μM for 3 h), fibroblasts (A and B) and iNeurons (C and D) were harvested and immunoblotted for the autophagic marker LC3 and against apoptotic markers PARP and CAS3. (E and F) Measurements of mitochondrial ROS production (E) and mitochondrial transmembrane potential (F) were studied in IDEB.-treated 11778 fibroblasts. (G–J) The same idebenone treatment was repeated in cybrids, where autophagy (G), apoptosis (H), ROS production (I), and mitochondrial transmembrane potential (J) were detected. Data are presented as means ± SEM. n = at least 3 independent experiments for western blots or 5 visual fields per at least 3 independent samples per condition for fluorescent microscopy experiments. ^∗^p < 0.05, ^∗∗^p < 0.01, and ^∗∗∗^p < 0.001.

Another therapeutic strategy in LHON is the administration of redox modulators, such as the short-chain benzoquinone idebenone (Gueven et al., 2021). Currently, idebenone is the only disease-specific drug approved by the European Medicine Agency (EMA) for LHON treatment (Amore et al., 2020). We tested idebenone, in its reduced form that exerts the therapeutic effect (Yu-Wai-Man et al., 2017), in LHON-affected fibroblasts (Figures 6A and 6B) and idNeurons (Figures 6C and 6D), observing a marked reduction of autophagy activity and apoptotic death in the LHON-affected cells. Idebenone administration also resulted in a concomitant reduction of ROS production (Figure 6E) and Ψ_m_ (Figure 6F). All these results were also confirmed in the cybrid cell model (Figures 6G–6J). Again, we did not observe any idebenone-related cytotoxic effect in control fibroblasts and cybrids (Figure S4I).

Finally, increased mitochondrial biogenesis has been documented to be a key spontaneous compensatory program activated in LHON-carriers, contributing to their lifelong unaffected state and incomplete penetrance (Giordano et al., 2014). We assessed protein expression of mitochondrial transcription factor A (TFAM) and cytochrome c oxidase (COX)-IV subunit as markers of mitochondrial mass in *ex*-*vivo*-collected PBMCs and in fibroblasts from a pair of discordant brothers carrying the 11778 mutation. Our results re-confirmed that LHON-carriers are the most efficient in compensatory mitobiogenesis (Figures S5A and S5B). Experiments aimed at detecting the amount of mtDNA confirmed the immunoblot results on mitochondrial mass (Figure S5C). Consistently, LHON-carrier fibroblasts also exhibited increased amounts of the “master regulator” of mitochondrial biogenesis peroxisome proliferator-activated receptor gamma, coactivator 1 α (PGC1-α) (Scarpulla, 2011) compared with LHON-affected samples (Figure S5D). We then asked whether this compensatory biogenesis could re-establish an optimal mitochondrial turnover and, as consequence, a healthy and functional mitochondrial pool in LHON-carriers. To visualize mitochondrial turnover, we used the fluorescent protein MitoTimer, whose fluorescence shifts from green to red as the mitochondrial population ages (Hernandez et al., 2013). The fluorescence analysis demonstrated that mitochondria present in LHON-affected fibroblasts were older and less functional than mitochondria of control fibroblasts, as well as of LHON-carrier fibroblasts (Figure S5E). Differently, cybrids generated from the same pair of LHON discordant brothers had comparable amounts of TFAM, COX-IV, and PGC1-α proteins and mtDNA content (Figures S5F and S5G), indicating that the crosstalk between mtDNA and specific nuclear backgrounds drives the compensation in LHON-carrier individuals. Noticeably, the 11778 mutation in the osteosarcoma nuclear background seems to have an opposite effect on mtDNA content compared with fibroblasts, since LHON-affected cybrids showed significantly lower mtDNA levels compared with wild-type cybrids, as previously observed in Giordano et al., 2011. Moreover, LHON-carrier cybrids displayed an age of mitochondrial population (Figure S5H) similar to that of LHON-affected cybrids, being both significantly older than in controls.

We thus evaluated whether an increase of mitobiogenesis might re-establish mitochondrial homeostasis in LHON cybrids. Overexpression of PGC1-α increased mitochondrial mass (Figure S6A) and lowered ROS production (Figure S6B) and Ψ_m_ (Figure S6C), reducing LC3 lipidation (Figure S6D) and protecting against apoptosis (Figure S6E).

## Discussion

The current study provides compelling evidence of a profound deregulation that targets autophagy removal of mitochondria, occurring in LHON-affected cell models, under basal conditions of cell culture, as well as in *ex vivo* LHON patient serum. This implicates a cellular stress phenotype documented by increased ROS production and Ψ_m_, ultimately resulting in a propensity toward apoptosis, undermining cell viability. We also show that this phenotype may be corrected by differently targeted therapeutic strategies including autophagy inhibitors acting at different stages of the autophagy mechanism (Choi et al., 2013; Park et al., 2012), redox modulators such as the EMA-approved idebenone (Amore et al., 2020; Gueven et al., 2021), and by genetically activating the mitobiogenesis program overexpressing the master regulator PGC1-α (Scarpulla, 2011). Individually, each of these therapeutic strategies was effective in our cellular models. Remarkably, all our experiments indicated that asymptomatic LHON-carriers have a naturally occurring compensation, which appears to be tightly dependent on the individual nuclear background, as this phenotype vanishes in cybrids. Ultimately, this compensatory phenotype makes LHON-carriers close to controls in all readouts analyzed despite the same LHON homoplasmic mutation as affected maternal relatives. The higher PGC1-α expression that we found only in LHON cells carrying the carrier nuclear background promotes mitochondrial biogenesis, allowing for alternative routes bypassing complex I impairment, as well as increasing the antioxidant machinery, globally compensating for the impaired OXPHOS (Giordano et al., 2014). Furthermore, the activation of the PPAR-γ/PGC1-α pathway through the induction of uncoupling protein 2 (UCP2) may also impinge on mitochondrial decoupling, reducing Ψ_m_ and ROS production and ultimately limiting oxidative damage and propensity to undergo apoptosis of LHON cells (Andrews et al., 2005). To reach such compensation, we envisage a better maintenance of mitochondrial homeostasis by shifting toward prevalent mitobiogenesis, which efficiently counterbalances the autophagy and mitophagy excessive activity determined by the mutant mtDNA. This highlights how these spontaneous compensatory mechanisms in RGCs, possibly prompted by yet unelucidated modifying nuclear genetic factors, are key to further refine the pathways that can be targeted therapeutically.

Our understanding that autophagy pathways are highly regulated cellular mechanisms has progressed impressively (Bialik et al., 2018; Choi et al., 2013). Not surprisingly, abnormalities of both autophagy and mitophagy occur in many human diseases. Despite this, it remains unclear as to whether these mechanisms are adaptive or maladaptive in some human pathologies. In neurodegeneration, an excessive number of autophagosomes may turn toxic for neuronal cells (Choi et al., 2013). As shown by previous studies and current results, the quality control of mitochondria is deeply integrated into a larger homeostatic program, balancing mitophagy and mitobiogenesis (Carelli et al., 2015a), strictly regulated by sensing OXPHOS efficiency and demand (Mishra et al., 2014). This in turn is tightly related to ROS production and retrograde signaling systems, key to coordinating the crosstalk of mtDNA and nuclear genome (Quiros et al., 2016). In LHON, this crosstalk may lead to very different outcomes as exemplified by the asymptomatic LHON-carriers when compared with LHON-affected brothers, which likely is determined by the context of the nuclear genetic background, lost in cybrids where only the mtDNA-related phenotype is transferred. This different cellular fate, previously centered only on the efficiency of mitochondrial biogenesis (Giordano et al., 2011, 2014), is now further elucidated by our current findings taking also into account autophagy and mitophagy. Their deregulation, in particular the increased basal levels of mitophagy and reduced reservoir to further activate this quality control program, as after starvation, becomes counterproductive for the neuronal homeostasis in RGCs. Recently, modeling of optic atrophy type 1 (OPA1) deficiency in mouse, worms, and cells demonstrated the detrimental impact that excessive autophagy and mitophagy may have on neuronal architecture and suggested that an increased removal of mitochondria, particularly at the axonal hillock, depletes axons and synapses of these organelles, leading to neurodegeneration (Zaninello et al., 2020). Remarkably, our results from LHON idNeurons show a similar phenotype, with mitochondria undergoing excessive mitophagy in the soma. The increase of basal autophagy and mitophagy is a common theme in mitochondrial diseases, in particular in those with optic atrophy, as documented by others and our own studies of different mtDNA mutations affecting ND subunits of complex I (Dombi et al., 2016; Granatiero et al., 2016), and OPA1-related syndromes (Carelli et al., 2015b; Liao et al., 2017). Only one study reported results partially in contrast to this. It suggested a reduced activation of the autophagy program in LHON cybrids under stress conditions and that rapamycin was able to correct the pathologic phenotype of LHON cells (Sharma et al., 2019). The experimental design in this study differed from ours, as their data were gathered in a stress model of LHON cybrids elicited by galactose as carbon source. Intriguingly, this study reported a higher content in lysosomes of LHON cybrids in glucose culture conditions, which actually mirrors our increased autophagy and mitophagy basal levels of LHON-affected cells compared with controls, and LHON-carriers.

It remains challenging to clarify the mechanism leading to the observed autophagy dysregulation in LHON. All common LHON pathogenic mutations (11778, 3460, and 14484) impair complex I-driven ATP synthesis (Baracca et al., 2005). This was also assessed *in vivo* by phosphorus-31 magnetic resonance spectroscopy of the maximum rate of ATP production in skeletal muscle (Lodi et al., 1997). Yet, the precise biochemical consequences of LHON pathogenic mutations remain controversial (Carelli et al., 2004; Fiedorczuk and Sazanov, 2018; Yu-Wai-Man et al., 2011). There are likely multiple mechanisms to bypass the complex I impairment. The prevalent pathological mechanism in LHON is currently ascribed to increased ROS production rather than a failure of bioenergetics (Beretta et al., 2004; Floreani et al., 2005; Lin et al., 2012). Furthermore, the paradoxical increase of Ψ_m_ has been explained by the low levels of complex II-driven O_2_ consumption and may be prevented by coinhibition of complex II, III, or IV activity (Forkink et al., 2014). Remarkably, a deregulation in Ca^2+^ homeostasis was also found in LHON, as in other mitochondrial diseases (Haroon et al., 2007; Wong and Cortopassi, 1997), providing another mechanism affecting the energetic balance of cells. Either mitochondrial Ca^2+^ accumulation (Danese et al., 2017; Giorgi et al., 2018a) or a decrease in ATP/ADP ratio, as well as an increased production of superoxide (O_2_^**.**^) and hydrogen peroxide (H_2_O_2_), the two major ROS species, can lead to AMPK induction (Rabinovitch et al., 2017; Zmijewski et al., 2010), which ultimately orchestrates the stress response (Toyama et al., 2016). In fact, we found an increase in the active form of AMPK, strongly suggestive that the AMPK pathway is instrumental to re-establish the correct levels of ATP. Remarkably, AMPK is also strictly linked to the execution of autophagy and mitophagy (Giorgi et al., 2018a). In particular, AMPK phosphorylation of ULK1 is required to activate autophagy and targeting of mitochondria to lysosomes. Compatibly, we found in LHON-affected cells an increased concentration of the pro-autophagy phosphorylated form of ULK1, associated with excessive autophagy levels and mitochondrial removal. Our results link the AMPK sensing of cellular bioenergetic impairment and ROS overproduction in LHON with the activation of autophagy and mitophagy.

The pathogenic mechanism here delineated may shed light on the massive and near synchronous death of RGCs, which occurs in LHON (Carelli et al., 2004; Yu-Wai-Man et al., 2011). There is mounting evidence that associates the autophagy pathways with cell-death programs (Galluzzi et al., 2018; Tsujimoto and Shimizu, 2005). In the RGCs, this translates into excessive levels of mitophagy that may become lethal for neuronal cells (Zaninello et al., 2020). How RGCs’ sudden decompensation occurs during the conversion from asymptomatic LHON-carrier to -affected, generating a wave of rapidly propagating cell death that characterizes the subacute phase of LHON (Coussa et al., 2019; Pan et al., 2012; Sadun et al., 2000), remains under scrutiny.

Fine-tuning of the balance between mitobiogenesis and mitophagy possibly distinguishes LHON-affected from -carriers and might also be the key in cases of therapeutic success. We showed that genetically boosting mitochondrial biogenesis by overexpressing PGC1-α rescued the mitochondrial stress phenotype, ultimately balancing autophagy and limiting apoptosis. Interestingly, idebenone, known for bypassing complex I, thus reducing ROS production and restoring downstream respiration, similarly mitigated autophagy and limited apoptosis. On the opposite side of the mitochondrial homeostatic balance, therapeutic agents directly hampering the autophagic activity by different mechanisms, such as 3-MA, CQ, and CL, resulted in a similar protective effect.

In conclusion, we provide convincing evidence of a comprehensive mechanism for LHON implicating a stress phenotype that results from altered balance of mitochondrial biogenesis and the quality control cycle. This is mediated by AMPK sensing of the primary biochemical defect that comes from complex I dysfunction and results in lowered bioenergetics efficiency combined with increased ROS levels, ultimately leading to increased propensity to apoptosis. This was evident in primary patient-derived cells such as fibroblasts but also transferred to cybrids, certifying the driving role of mtDNA LHON mutations, and, for the first time, was demonstrated in idNeurons, indicating the relevance of this mechanistic pathway in the cell type targeted in LHON. Remarkably, the compensatory phenotype displayed by LHON-carriers was shown to be driven by the nuclear individual background, as was lost in cybrid experiments. This compensatory phenotype derives from prevalent mitobiogenesis counteracting excessive mitophagy and globally rebalancing the stress phenotype. Finally, we provided proof of principle that manipulating, genetically or pharmacologically, this mechanistic pathway efficiently corrects the key readouts of pathology. These results cast hope for a rapid translation into clinical trials with LHON patients, as many of the drugs proposed can be re-purposed to exploit these therapeutic strategies.

## STAR★Methods

### Key resources table

**Table:** 

REAGENT or RESOURCE	SOURCE	IDENTIFIER
**Antibodies**		

anti-LC3B	Sigma Aldrich	CST: 2775; RRID: AB_796155
anti-LC3B	Novus Biological	NB100-2220; RRID: AB_10003146
anti-GAPDH	Cell Signaling	CST: 2118; RRID: AB_561053
anti-GAPDH	Sigma Aldrich	G8795; RRID: AB_1078991
anti-TUBB	Sigma Aldrich	T5201; RRID: AB_609915
anti-ACTN	Sigma Aldrich	A1978; RRID: AB_476692
anti-p62/SQSTM1	Sigma Aldrich	P0067; RRID: AB_1841064
anti- βIII-Tub	Thermo Fisher Scientific	MA1-118; RRID: AB_2536829
anti-ACC	Cell Signaling	CST: 3662; RRID: AB_2219400
anti-p-ACC	Cell Signaling	CST: 11818; RRID: AB_2687505
anti-ULK1	Cell Signaling	CST: 8054; RRID: AB_11178668
anti-p-ULKser317	Cell Signaling	CST: 12753; RRID: AB_2687883
anti-AMPK	Cell Signaling	CST: 2352; RRID: AB_330331
anti-p-AMPK	Cell Signaling	CST: 2531; RRID: AB_330330
anti-4EBP1	Thermo Fisher Scientific	AHO1382; RRID: AB_2536336
anti-p-4EBP1	Thermo Fisher Scientific	700238; RRID: AB_2532302
anti-PARP	Cell Signaling	CST: 9542; RRID: AB_2160739
anti-CASP3	Cell Signaling	CST: 9662; RRID: AB_331439
anti-PGC1 α	Thermo Fisher Scientific	PA5-38021; RRID: AB_2554625
anti-COXIV	Thermo Fisher Scientific	20E8C12; RRID: AB_2535839
anti-TFAM	Abcam	ab47517; RRID: AB_945799
anti-NEUN	Millipore	MAB377; RRID: AB_2298772
anti-MAP2	Cell Signaling	CST: 4542S; RRID: AB_10693782
anti- βIII-Tub	GenScript	A01627; RRID: AB_2622164

**Chemicals, peptides, and recombinant proteins**		

Clozapine	Sigma Aldrich	C6305
Chloroquine	Sigma Aldrich	C6628
3-methyladenine	Sigma Aldrich	M9281
bafilomycin A1	Sigma Aldrich	B1793
Idebenone	Sigma Aldrich	I5659
Lipofectamine reagent	Thermo Fisher Scientific	15338100
MitoTracker Green FM	Thermo Fisher Scientific	M7514
LysoTracker Red DND-99	Thermo Fisher Scientific	L7528
MitoSOX Red	Thermo Fisher Scientific	M36008
Tetramethylrhodamine, Methyl Ester, Perchlorate (TMRM)	Thermo Fisher Scientific	T-668

**Critical commercial assays**		

ELISA KIT ATG5	My Biosource	MS7209535
ELISA KIT Parkin	My Biosource	MBS732278
ELISA KIT Optineurin	My Biosource	MBS2704623
ELISA KIT ATG7	My Biosource	MBS062423
NucleoSpin Tissue	Macherey-Nagel	REF 740952.50

**Experimental models: Cell lines**		

Detailed information of cell used in this Research Article are reported in Table S1 and in STAR Methods at the section experimental model and subject details	N/A	N/A

**Recombinant DNA**		

GFP-LC3	(Patergnani et al., 2013)	N/A
mCherry-GFP-LC3	(Patergnani et al., 2013)	N/A
YFP-Parkin	Addgene	Plasmid #23955; RRID: Addgene_23955
mitochondrial (mt)-Cherry	Addgene	Plasmid #55102; RRID: Addgene_55102
pTRE-tight-MITO TIMER	Addgene	plasmid # 50547; RRID: Addgene_50547

**Software and algorithms**		

Graphpad Prism v9.3.1	GraphPad	RRID: SCR_002798
FiJi (ImageJ)	https://imagej.net/software/fiji/	RRID: SCR_002285
EndNoteX9	EndNote	RRID: SCR_014001
Biorender	https://biorender.com/	RRID: SCR_018361

### Resource availability

#### Lead contact

Further information and requests for resources and reagents should be directed to and will be fulfilled by the Lead Contact: Paolo Pinton (paolo.pinton@unife.it).

#### Materials availability

This work did not generate any unique reagents.

### Experimental model and subject details

#### Human samples

LHON patients, initially diagnosed by restriction fragment length polymorphism (RFLP) analysis, and healthy individuals were recruited at the IRCCS Istituto delle Scienze Neurologiche di Bologna (Bologna, Italy) for blood samples collection. The study is part of a research project approved by the institutional ethical board (Comitato Etico di Area Vasta Emilia Centro-CE-AVEC, code CE 19072), and all participants gave informed written consent. All procedures were performed according to the Declaration of Helsinki. Fibroblasts cell lines were previously generated as part of a research project (Comitato Etico dell’Azienda Ospedaliero-Universitaria di Bologna, Policlinico Sant’Orsola Malpighi, number 123/2006/U/Sper). The generation of LHON iPSCs was approved by the Institutional ethical board (number 43-2013, date 26/6/2013) of Fondazione IRCCS Istituto Neurologico Carlo Besta.

#### Fibroblasts

Fibroblast lines were established from skin biopsies of four control individuals (two males of 22 and 35 years, two females of 29 and 32 years), three affected patients (two m.11778G>A males of 36 and 55 years, and one m.3460G>A female of 19 years) and two unaffected mutation-carriers (m.11778G>A males of 38 and 46 years), the respective brothers of the affected patients with the same mutation (see Table S1). Cells were grown in DMEM (EuroClone, Milano, Italy) containing 25 mM glucose supplemented with 10% fetal bovine serum (FBS), 2 mM L-glutamine, 100 U/mL penicillin and 100 μg/mL streptomycin and were maintained at 37°C in a humidified atmosphere with 5% CO_2._ Functional experiments were carried out on sub-confluent cell cultures with a comparable number of passages (10–20).

#### Induced pluripotent stem cells (iPSC)

Transgene-free induced pluripotent stem cells (iPSC) were generated, from control (male of 34 years) and two affected patient fibroblasts (one m.3460G>A female of 19 years and one m.11778G>A male of 26 years), as previously described (Peron et al., 2020). Briefly, iPSC were generated by a CytoTune-iPS 2.0 Sendai Reprogramming Kit (Orellana et al., 2016), introducing the four transcription factors proposed by Yamanaka: OCT4, SOX2, KLF4 and c-MYC. Pluripotency of iPSC was characterized by alkaline phosphatase, PCR and immunofluorescence for the pluripotent markers Sox2, Rex1, Tra1-60, Nanog and Oct4. Integrity of nuclear and mitochondrial genomes was verified by Comparative Genomic Hybridization (CGH) array (Galizia et al., 2012) and complete mtDNA sequence by NGS, as previously described (Caporali et al., 2018).

#### Cybrids cell lines

Cybrid cell lines were generated as previously described in (Cock et al., 1998; Floreani et al., 2005; Ghelli et al., 2003; Zanna et al., 2005) by using enucleated fibroblasts as the mitochondria donor, and from the osteosarcoma (143B.TK−)-derived 206 cell line (female, 13 years), as the acceptor rho0 cell line (see Table S1). Cybrid cell lines were grown in DMEM supplemented with 10% FBS, 2 mM L-glutamine, 100 units/mL penicillin, 100 μg/mL streptomycin, and 0.1 mg/mL bromodeoxyuridine. For experiments, cells were seeded at 4 x 10^5^ cells/cm2 and incubated in DMEM containing 25 mM glucose supplemented with 10% FBS, 2 mM L-glutamine, 100 U/mL penicillin and 100 μg/mL streptomycin at 37°C in an incubator with a humidified atmosphere of 5% CO_2_.

#### Peripheral blood cells isolation and processing

Ten ml of venous blood was collected in EDTA from 17 healthy controls (8 females and 9 males, with average age 47.5 years (SD = 14.5) and 38.5 years (SD = 13.9), respectively), 10 LHON-affected (8 males with average age 38.8 years (SD = 11.6) and two females of age 49 and 59 years) and 14 LHON-carriers patients, all carrying the 11778 homoplasmic mutation (7 females and 7 males, with average age 46.4 years (SD = 10.7) and 26.1 years (SD = 5.9), respectively). PBMCs were isolated using a density gradient cell separation medium (Sigma Aldrich: Histopaque-1077), following manufacturer’s instructions. Proteins were extracted from PBMCs with RIPA buffer containing proteases inhibitors cocktail (Roche: 11697498001), following standard procedures, and protein content was assessed with Bradford method.

#### Serum levels of ATG5, ATG7, optineurin and parkin determination

Peripheral blood from ten healthy controls (3 females and 7 males, with average age 37.6 years (SD = 17.5) and 38.1 years (SD = 15.9) respectively), seven LHON-affected (1 female of 59 years and 6 males, with average age 39 years (SD = 21.8)) and nine LHON-carriers (4 females and 5 males, with average age 35.5 years (SD = 17.3) and 37.2 years (SD = 15.5) respectively), all carrying the 11778 homoplasmic mutation, was collected in Serum Separation Tubes and centrifuged 15 min at 2900 x g. Serum samples were aliquoted and stored at -80°C until processing. Concentrations of ATG5, ATG7, Parkin and Optineurin were determined by using commercially available enzyme-linked immunosorbent assay (ELISA) kits (My Biosource, San Diego, California, USA; MS7209535 for ATG5, MBS062423 for ATG7, MBS732278 for Parkin and MBS2704623 for Optineurin) following the manufacturer’s instructions as previously published (Patergnani et al., 2018).

### Method details

#### Immunoblotting

For immunoblotting, cells were scraped into ice-cold phosphate–buffered saline and lysed in modified 10 mM Tris buffer (pH 7.4) containing 150 mM NaCl, 1% Triton X-100, 10% glycerol, 10 mM EDTA and protease inhibitor cocktail. After lysis on ice, homogenates were cleared via centrifugation at 12,000 g at 4°C for 10 min. Protein extracts were quantified using the Lowry assay (Bio-Rad Laboratories).

Protein extracts (20 μg for cells and 30 ug for PBMCs) were separated on 4–12% or 12% bis-Tris acrylamide gels (Life technologies: NP0323, EC6026, and NP0341) and electrotransferred to PVDF or nitrocellulose membrane according to standard procedures. Nonspecific binding sites were saturated by incubating membranes with TBS-Tween 20 (0.05%) supplemented with 5% non-fat powdered milk for 1 h. Next, membranes were incubated overnight with primary antibodies and then were assessed with appropriate HRP-labeled secondary antibodies and chemiluminescent substrate, or with fluorescent secondary antibodies.

#### Mitochondrial DNA content assessment and next generation sequencing

Total DNA was isolated from cell pellets using the commercial kit NucleoSpin Tissue (Macherey-Nagel, REF 740952.50). MtDNA quantification was performed by a Real Time-PCR assay based on hydrolysis probe chemistry previously used (Giordano et al., 2014). Briefly, an mtDNA fragment (*MT-ND2* gene) and a nuclear DNA fragment (*FASLG* gene) were coamplified by multiplex PCR, and their concentration was determined by absolute quantification through a standard curve made with serial dilutions of a plasmid containing a copy of the two amplicons. Primers, probes, and conditions have been previously published (Mussini et al., 2005). The presence of LHON homoplasmic mutations, in both fibroblasts and cybrids, was assessed by complete mtDNA sequencing as previously reported (Caporali et al., 2018). The protocol consists in two overlapping long PCR amplicons (9.1 kb and 11.2 kb), amplified with PrimeSTAR Max DNA Polymerase (Takara), following the manufacture instructions. The library was constructed by xGen DNA Lib Prep EZ (IDT) and sequenced on MiSeq System (Illumina). Reads were aligned to the human reference mitochondrial genome (NC_012920.1) and variants were called by Mitoverse. The mtDNA haplogroup affiliations were assigned using HaploGrep2.4.0, according to PhyloTree Build 17 (www.phylotree.org). MtDNA haplogroups and private variants for each cell line are reported in Table S1.

#### Fluorescence microscopy and quantitative analysis of GFP-LC3 puncta

Fibroblast and cybrid cells were cultured on 24-mm glass coverslips and, at 50% confluence, were transfected with Lipofectamine reagent (Thermo Fisher Scientific: 15338100) and 1 μg of plasmid DNA (GFP-LC3). After 36 h, images were taken on a Nikon LiveScan Swept Field Confocal Microscope (SFC) Eclipse Ti equipped with NIS-Elements microscope imaging software and on a confocal laser scanning microscopy Olympus FV3000 both equipped with a 63× oil immersion objective (N.A. 1.4). For each condition, the GFP-LC3 puncta were counted in at least 25 independent visual fields.

#### Mitophagy assessment with LysoTracker Red and MitoTracker Green

Mitophagy experiments were performed in fibroblasts, cybrids and 35 days old idNeurons. Cells were incubated with MitoTracker Green FM (1 μM final concentration) (Thermo Fisher Scientific: M7514) for 30 min at 37°C and then extensively washed with PBS. LysoTracker Red DND-99 (1 μM final concentration) (Thermo Fisher Scientific: L7528) was then added, and cells were immediately observed on a Nikon LiveScan Swept Field Confocal Microscope (SFC) Eclipse Ti equipped with NIS-Elements microscope imaging software and on a confocal laser scanning microscopy Olympus FV3000 both equipped with a 63× oil immersion objective (N.A. 1.4). The green and red signal colocalization rate was evaluated using the colocalization counter JACOP available in Fiji software. For each condition, the colocalization of these two signals was also determined by manual counting of fluorescent puncta. For the region-specific mitophagy levels into idNeurons, Region Of interest (ROI) of soma and axon-dendrites regions, considering the entire length of the latter, were created and analyzed by the colocalization plug-in Coloc2 available in Fiji software. For each ROI the Manders’ parameter was calculated.

#### Mitophagy assessment with YFP-Parkin and mitochondrial (mt)-Cherry

Fibroblasts and cybrid cells were cultured on 24-mm glass coverslips and, at 50% confluence, were transfected with Lipofectamine reagent (Thermo Fisher Scientific: 15338100) and 2 μg of plasmid DNA (1 μg YFP-Parkin (Addgene plasmid # 23955) (Narendra et al., 2008) and 1 ug mt-Cherry (Addgene plasmid # 55102) (Olenych et al., 2007)). After 36 h, images were taken on a confocal laser scanning microscopy Olympus FV3000 equipped with a 63× oil immersion objective (N.A. 1.4). The number of Parkin dots on mitochondria per cell and the representative images were generated by using NIH ImageJ software.

#### MitoTimer measurements

Cells were transfected with the pTRE-tight-MITO TIMER plasmid (Addgene plasmid # 50547) (Hernandez et al., 2013). After 36h, cells were imaged using excitation at 490 nm and 550 nm and emission of green (500–540 nm) and red (580–640 nm) fluorescence signals by using a Zeiss LSM510 confocal microscope and an Olympus scanning microscope equipped of 63× oil immersion objective (N.A. 1.4). The ratio of the fluorescence signal intensity in the red and green channels and the representative images were generated by using NIH ImageJ software as previously reported in (Morciano et al., 2021).

#### Autophagy induction and inhibition

The autophagy process *in vitro* was triggered through serum deprivation (EBSS, 30 min). The pharmacological inhibition of autophagy was performed by treating cells with 3-MA, (Merck: M9281) (2,5 μM), CL (Merck: C6305) (1 μM) or CQ (Merck: C6628) (1 μM) in DMEM supplemented with 10% FBS. After treatment, cells were fixed or lysed to detect the amount of autophagosome vesicles by fluorescence microscopy with immunoblot analysis (using an anti-LC3 antibody).

#### mROS measurements

Total ROS release from mitochondria was estimated fluorometrically by MitoSOX Red probe oxidation (Thermo Fisher and Tali™ Image-Based Cytometer). Fluorescence was measured using 510 ± 10 nm and 595 ± 35 nm excitation and emission wavelengths, respectively.

#### Measurement of Ψ_m_

The Ψ_m_ was measured by labeling cells with 20 nM TMRM (Life Technologies: T-668) for 30 min at 37°C. Images were taken on an inverted microscope (Nikon LiveScan Swept Field Confocal Microscope (SFC) Eclipse Ti equipped of with Elements microscope imaging software). TMRM was excited at 560 nm, and the emission signal was collected through a 590–650-nm bandpass filter. Images were taken every 5 s with a fixed 20 ms exposure time. Carbonyl cyanide p- trifluoromethoxyphenylhydrazone (FCCP, 10 μM), an oxidative phosphorylation uncoupler, was added after 12 acquisitions to completely collapse the electrical gradient established by the respiratory chain.

#### Cell proliferation and viability assay

Cells were seeded at 15,000 cells per well in 6-well plates. Cells were seeded on 5 plates; one plate for each day. Every 24 h after seeding, cells were washed once with PBS and fixed in 4% paraformaldehyde in PBS for 15 min. Cells were stained with 0.1% crystal violet for 20 min and then washed thrice with water. To each well, 500 μL of 10% acetic acid was added, and the cells were incubated for 20 min with shaking (extraction). Absorbance was measured at 590 nm.

#### Cell transfection

Where indicated, cybrid cell lines were transfected with Lipofectamine reagent (Thermo Fisher, 15338100) and 1 μg of plasmid DNA. Cells were analyzed after at least 36 h of expression.

#### Neural precursor cells (NPC)

NPCs were obtained by multiple steps. Embryoid body suspensions were cultured for 5 days and then plated on Matrigel-coated plates to generate neuro-ectodermal rosette structures. After 5-7 days, rosettes were picked and plated on polyornithine/laminin to generate and maintain NPC cultures [modified from (Brafman, 2015)]. NPCs were characterized after passage 3, by PCR and immunofluorescence for expression of neural precursor markers (Sox1, Pax6, Nestin) and absence of pluripotent markers (Oct4 and Nanog).

To obtain iPSC-derived neurons (idNeurons), NPCs were plated on glass supports in polyornithine/laminin-coated plates. The next day, neuronal differentiation medium (DMEM/F-12, 1× N2 supplement, 1× B27 supplement, 30 ng/mL BDNF, 30 ng/mL GDNF, 1 μM DAPT, 1× penicillin/streptomycin, 1× glutamine and 1× non-essential amino acids) was added to the cells and then was changed every other day. After 35 days, the idNeurons were characterized and utilized in all the experiments here reported. Characterization was performed by immunofluorescence to verify the specific expression of typical neuronal markers such as NEUN (Millipore, MAB377), MAP2 (Cell Signaling, #4542S) and βIII-TUBULIN (GenScript, A01627). The following dilutions were used: anti-NEUN 1:1000; anti-MAP2 1:200; anti-βIII TUBULIN 1:500.

#### Quantitative analysis of autophagy flux

LHON fibroblasts cultured on 24-mm glass coverslips were transfected with Lipofectamine reagent (Thermo Fisher Scientific: 15338100) and 1 μg of plasmid DNA (mCherry-eGFP-LC3). After 36 h of transfection, cells were imaged at 60× magnification with a Nikon LiveScan Swept Field Confocal Microscope (SFC) Eclipse Ti equipped with NIS-Elements microscope imaging software. Obtained puncta images were merged to compare RFP and GFP signals using ImageJ software. For each condition, the colocalization of these two signals was determined by manual counting of fluorescent puncta in at least 20 independent visual fields.

### Quantification and statistical analysis

All data were analyzed by Prism 8 (GraphPad Software Inc.). Unless otherwise specified, data are representative of at least three biologically independent experiments. The comparison between three groups by means of the one-way ANOVA test followed by Tukey’s multiple comparisons test, while the comparison between two groups using the unpaired t test of Student. p values < 0.05 were considered statistically significant and marked with asterisks (^∗^p < 0.05; ^∗∗^p < 0.01; ^∗∗∗^p < 0.001), as indicated in Figure legends. All data collected are represented as mean ± s.e.m. The p values of histograms that did not reach statistical significance in any of the conditions examined are reported in Table S2.

## Data Availability

This paper does not report original code. All software utilized is freely or commercially available and is listed in the key resources table. All data reported in this paper will be shared by the lead contact upon request.

## Supplemental information

Document S1. Figures S1–S6 and Tables S1 and S2

Document S2. Article plus supplemental information

## References

[bib1] Amore G., Romagnoli M., Carbonelli M., Barboni P., Carelli V., La Morgia C. (2020). Therapeutic options in hereditary optic neuropathies. Drugs.

[bib2] Andrews Z.B., Diano S., Horvath T.L. (2005). Mitochondrial uncoupling proteins in the CNS: in support of function and survival. Nat. Rev. Neurosci..

[bib3] Baracca A., Solaini G., Sgarbi G., Lenaz G., Baruzzi A., Schapira A.H.V., Martinuzzi A., Carelli V. (2005). Severe impairment of complex I-driven adenosine triphosphate synthesis in leber hereditary optic neuropathy cybrids. Arch. Neurol..

[bib4] Bargiela D., Yu-Wai-Man P., Keogh M., Horvath R., Chinnery P.F. (2015). Prevalence of neurogenetic disorders in the North of England. Neurology.

[bib5] Beretta S., Mattavelli L., Sala G., Tremolizzo L., Schapira A.H.V., Martinuzzi A., Carelli V., Ferrarese C. (2004). Leber hereditary optic neuropathy mtDNA mutations disrupt glutamate transport in cybrid cell lines. Brain.

[bib6] Bialik S., Dasari S.K., Kimchi A. (2018). Autophagy-dependent cell death - where, how and why a cell eats itself to death. J. Cell Sci..

[bib7] Brafman D.A. (2015). Generation, expansion, and differentiation of human pluripotent stem cell (hPSC) derived neural progenitor cells (NPCs). Methods Mol. Biol..

[bib8] Caporali L., Iommarini L., La Morgia C., Olivieri A., Achilli A., Maresca A., Valentino M.L., Capristo M., Tagliavini F., Del Dotto V. (2018). Peculiar combinations of individually non-pathogenic missense mitochondrial DNA variants cause low penetrance Leber's hereditary optic neuropathy. PLoS Genet..

[bib9] Carelli V., d'Adamo P., Valentino M.L., La Morgia C., Ross-Cisneros F.N., Caporali L., Maresca A., Loguercio Polosa P., Barboni P., De Negri A. (2016). Parsing the differences in affected with LHON: genetic versus environmental triggers of disease conversion. Brain.

[bib10] Carelli V., Ghelli A., Ratta M., Bacchilega E., Sangiorgi S., Mancini R., Leuzzi V., Cortelli P., Montagna P., Lugaresi E., Degli Esposti M. (1997). Leber's hereditary optic neuropathy: biochemical effect of 11778/ND4 and 3460/ND1 mutations and correlation with the mitochondrial genotype. Neurology.

[bib11] Carelli V., Giordano C., d'Amati G. (2003). Pathogenic expression of homoplasmic mtDNA mutations needs a complex nuclear-mitochondrial interaction. Trends Genet..

[bib12] Carelli V., Maresca A., Caporali L., Trifunov S., Zanna C., Rugolo M. (2015). Mitochondria: biogenesis and mitophagy balance in segregation and clonal expansion of mitochondrial DNA mutations. Int. J. Biochem. Cell Biol..

[bib13] Carelli V., Musumeci O., Caporali L., Zanna C., La Morgia C., Del Dotto V., Porcelli A.M., Rugolo M., Valentino M.L., Iommarini L. (2015). Syndromic parkinsonism and dementia associated with OPA1 missense mutations. Ann. Neurol..

[bib14] Carelli V., Ross-Cisneros F.N., Sadun A.A. (2004). Mitochondrial dysfunction as a cause of optic neuropathies. Prog. Retin. Eye Res..

[bib15] Chen R., Zou Y., Mao D., Sun D., Gao G., Shi J., Liu X., Zhu C., Yang M., Ye W. (2014). The general amino acid control pathway regulates mTOR and autophagy during serum/glutamine starvation. J. Cell Biol..

[bib16] Choi A.M.K., Ryter S.W., Levine B. (2013). Autophagy in human health and disease. N. Engl. J. Med..

[bib17] Cock H.R., Tabrizi S.J., Cooper J.M., Schapira A.H. (1998). The influence of nuclear background on the biochemical expression of 3460 Leber's hereditary optic neuropathy. Ann. Neurol..

[bib18] Coussa R.G., Merat P., Levin L.A. (2019). Propagation and selectivity of axonal loss in leber hereditary optic neuropathy. Sci. Rep..

[bib19] Danese A., Patergnani S., Bonora M., Wieckowski M.R., Previati M., Giorgi C., Pinton P. (2017). Calcium regulates cell death in cancer: roles of the mitochondria and mitochondria-associated membranes (MAMs). Biochim. Biophys. Acta Bioenerg..

[bib20] Dombi E., Diot A., Morten K., Carver J., Lodge T., Fratter C., Ng Y.S., Liao C., Muir R., Blakely E.L. (2016). The m.13051G>A mitochondrial DNA mutation results in variable neurology and activated mitophagy. Neurology.

[bib21] Egan D.F., Shackelford D.B., Mihaylova M.M., Gelino S., Kohnz R.A., Mair W., Vasquez D.S., Joshi A., Gwinn D.M., Taylor R. (2011). Phosphorylation of ULK1 (hATG1) by AMP-activated protein kinase connects energy sensing to mitophagy. Science.

[bib22] Fiedorczuk K., Sazanov L.A. (2018). Mammalian mitochondrial complex I structure and disease-causing mutations. Trends Cell Biol..

[bib23] Floreani M., Napoli E., Martinuzzi A., Pantano G., De Riva V., Trevisan R., Bisetto E., Valente L., Carelli V., Dabbeni-Sala F. (2005). Antioxidant defences in cybrids harboring mtDNA mutations associated with Leber's hereditary optic neuropathy. FEBS J..

[bib24] Forkink M., Manjeri G.R., Liemburg-Apers D.C., Nibbeling E., Blanchard M., Wojtala A., Smeitink J.A.M., Wieckowski M.R., Willems P.H.G.M., Koopman W.J.H. (2014). Mitochondrial hyperpolarization during chronic complex I inhibition is sustained by low activity of complex II, III, IV and V. Biochim. Biophys. Acta.

[bib25] Galizia E.C., Srikantha M., Palmer R., Waters J.J., Lench N., Ogilvie C.M., Kasperavičiūtė D., Nashef L., Sisodiya S.M. (2012). Array comparative genomic hybridization: results from an adult population with drug-resistant epilepsy and co-morbidities. Eur. J. Med. Genet..

[bib26] Galluzzi L., Yamazaki T., Kroemer G. (2018). Linking cellular stress responses to systemic homeostasis. Nat. Rev. Mol. Cell Biol..

[bib27] Ghelli A., Zanna C., Porcelli A.M., Schapira A.H.V., Martinuzzi A., Carelli V., Rugolo M. (2003). Leber's hereditary optic neuropathy (LHON) pathogenic mutations induce mitochondrial-dependent apoptotic death in transmitochondrial cells incubated with galactose medium. J. Biol. Chem..

[bib28] Giordano C., Iommarini L., Giordano L., Maresca A., Pisano A., Valentino M.L., Caporali L., Liguori R., Deceglie S., Roberti M. (2014). Efficient mitochondrial biogenesis drives incomplete penetrance in Leber's hereditary optic neuropathy. Brain.

[bib29] Giordano C., Montopoli M., Perli E., Orlandi M., Fantin M., Ross-Cisneros F.N., Caparrotta L., Martinuzzi A., Ragazzi E., Ghelli A. (2011). Oestrogens ameliorate mitochondrial dysfunction in Leber's hereditary optic neuropathy. Brain.

[bib30] Giordano L., Deceglie S., d'Adamo P., Valentino M.L., La Morgia C., Fracasso F., Roberti M., Cappellari M., Petrosillo G., Ciaravolo S. (2015). Cigarette toxicity triggers Leber's hereditary optic neuropathy by affecting mtDNA copy number, oxidative phosphorylation and ROS detoxification pathways. Cell Death Dis..

[bib31] Giorgi C., Bouhamida E., Danese A., Previati M., Pinton P., Patergnani S. (2021). Relevance of autophagy and mitophagy dynamics and markers in neurodegenerative diseases. Biomedicines.

[bib32] Giorgi C., Danese A., Missiroli S., Patergnani S., Pinton P. (2018). Calcium dynamics as a machine for decoding signals. Trends Cell Biol..

[bib33] Giorgi C., Marchi S., Pinton P. (2018). The machineries, regulation and cellular functions of mitochondrial calcium. Nat. Rev. Mol. Cell Biol..

[bib34] Granatiero V., Giorgio V., Calì T., Patron M., Brini M., Bernardi P., Tiranti V., Zeviani M., Pallafacchina G., De Stefani D., Rizzuto R. (2016). Reduced mitochondrial Ca(2+) transients stimulate autophagy in human fibroblasts carrying the 13514A>G mutation of the ND5 subunit of NADH dehydrogenase. Cell Death Differ..

[bib35] Gueven N., Ravishankar P., Eri R., Rybalka E. (2021). Idebenone: when an antioxidant is not an antioxidant. Redox Biol..

[bib36] Haroon M.F., Fatima A., Schöler S., Gieseler A., Horn T.F.W., Kirches E., Wolf G., Kreutzmann P. (2007). Minocycline, a possible neuroprotective agent in Leber's hereditary optic neuropathy (LHON): studies of cybrid cells bearing 11, 778 mutation. Neurobiol. Dis..

[bib37] Hernandez G., Thornton C., Stotland A., Lui D., Sin J., Ramil J., Magee N., Andres A., Quarato G., Carreira R.S. (2013). MitoTimer: a novel tool for monitoring mitochondrial turnover. Autophagy.

[bib38] Hirst J., Roessler M.M. (2016). Energy conversion, redox catalysis and generation of reactive oxygen species by respiratory complex I. Biochim. Biophys. Acta.

[bib39] Jiang P., Jin X., Peng Y., Wang M., Liu H., Liu X., Zhang Z., Ji Y., Zhang J., Liang M. (2016). The exome sequencing identified the mutation in YARS2 encoding the mitochondrial tyrosyl-tRNA synthetase as a nuclear modifier for the phenotypic manifestation of Leber's hereditary optic neuropathy-associated mitochondrial DNA mutation. Hum. Mol. Genet..

[bib40] Kanki T., Okamoto K. (2014). Assays for autophagy II: mitochondrial autophagy. Methods Mol. Biol..

[bib41] Katsuragi Y., Ichimura Y., Komatsu M. (2015). p62/SQSTM1 functions as a signaling hub and an autophagy adaptor. FEBS J..

[bib42] Kim J., Kundu M., Viollet B., Guan K.L. (2011). AMPK and mTOR regulate autophagy through direct phosphorylation of Ulk1. Nat. Cell Biol..

[bib43] King M.P., Attardi G. (1989). Human cells lacking mtDNA: repopulation with exogenous mitochondria by complementation. Science.

[bib44] King M.P., Koga Y., Davidson M., Schon E.A. (1992). Defects in mitochondrial protein synthesis and respiratory chain activity segregate with the tRNA(Leu(UUR)) mutation associated with mitochondrial myopathy, encephalopathy, lactic acidosis, and strokelike episodes. Mol. Cell Biol..

[bib45] Klionsky D.J., Abdel-Aziz A.K., Abdelfatah S., Abdellatif M., Abdoli A., Abel S., Abeliovich H., Abildgaard M.H., Abudu Y.P., Acevedo-Arozena A. (2021). Guidelines for the use and interpretation of assays for monitoring autophagy. Autophagy.

[bib46] Korsten A., de Coo I.F.M., Spruijt L., de Wit L.E.A., Smeets H.J.M., Sluiter W. (2010). Patients with Leber hereditary optic neuropathy fail to compensate impaired oxidative phosphorylation. Biochim. Biophys. Acta.

[bib47] Li L., Chen Y., Gibson S.B. (2013). Starvation-induced autophagy is regulated by mitochondrial reactive oxygen species leading to AMPK activation. Cell. Signal..

[bib48] Liao C., Ashley N., Diot A., Morten K., Phadwal K., Williams A., Fearnley I., Rosser L., Lowndes J., Fratter C. (2017). Dysregulated mitophagy and mitochondrial organization in optic atrophy due to OPA1 mutations. Neurology.

[bib49] Lin C.S., Sharpley M.S., Fan W., Waymire K.G., Sadun A.A., Carelli V., Ross-Cisneros F.N., Baciu P., Sung E., McManus M.J. (2012). Mouse mtDNA mutant model of Leber hereditary optic neuropathy. Proc. Natl. Acad. Sci. USA.

[bib50] Mishra P., Carelli V., Manfredi G., Chan D.C. (2014). Proteolytic cleavage of Opa1 stimulates mitochondrial inner membrane fusion and couples fusion to oxidative phosphorylation. Cell Metab..

[bib51] Missiroli S., Bonora M., Patergnani S., Poletti F., Perrone M., Gafà R., Magri E., Raimondi A., Lanza G., Tacchetti C. (2016). PML at mitochondria-associated membranes is critical for the repression of autophagy and cancer development. Cell Rep..

[bib52] Mizushima N., Yoshimori T., Levine B. (2010). Methods in mammalian autophagy research. Cell.

[bib53] Morciano G., Patergnani S., Pedriali G., Cimaglia P., Mikus E., Calvi S., Albertini A., Giorgi C., Campo G., Ferrari R., Pinton P. (2021). Impairment of mitophagy and autophagy accompanies calcific aortic valve stenosis favoring cell death and the severity of disease. Cardiovasc. Res..

[bib54] Mussini C., Pinti M., Bugarini R., Borghi V., Nasi M., Nemes E., Troiano L., Guaraldi G., Bedini A., Sabin C. (2005). Effect of treatment interruption monitored by CD4 cell count on mitochondrial DNA content in HIV-infected patients: a prospective study. AIDS.

[bib55] Narendra D., Tanaka A., Suen D.F., Youle R.J. (2008). Parkin is recruited selectively to impaired mitochondria and promotes their autophagy. J. Cell Biol..

[bib56] Narendra D.P., Jin S.M., Tanaka A., Suen D.F., Gautier C.A., Shen J., Cookson M.R., Youle R.J. (2010). PINK1 is selectively stabilized on impaired mitochondria to activate Parkin. PLoS Biol..

[bib57] Olenych S.G., Claxton N.S., Ottenberg G.K., Davidson M.W. (2007). The fluorescent protein color palette. Curr. Protoc. Cell Biol..

[bib58] Orellana D.I., Santambrogio P., Rubio A., Yekhlef L., Cancellieri C., Dusi S., Giannelli S.G., Venco P., Mazzara P.G., Cozzi A. (2016). Coenzyme A corrects pathological defects in human neurons of PANK2-associated neurodegeneration. EMBO Mol. Med..

[bib59] Pan B.X., Ross-Cisneros F.N., Carelli V., Rue K.S., Salomao S.R., Moraes-Filho M.N., Moraes M.N., Berezovsky A., Belfort R., Sadun A.A. (2012). Mathematically modeling the involvement of axons in Leber's hereditary optic neuropathy. Invest. Ophthalmol. Vis. Sci..

[bib60] Park J., Chung S., An H., Kim J., Seo J., Kim D.H., Yoon S.Y. (2012). Haloperidol and clozapine block formation of autophagolysosomes in rat primary neurons. Neuroscience.

[bib61] Patergnani S., Bonora M., Bouhamida E., Danese A., Marchi S., Morciano G., Previati M., Pedriali G., Rimessi A., Anania G. (2021). Methods to monitor mitophagy and mitochondrial quality: implications in cancer, neurodegeneration, and cardiovascular diseases. Methods Mol. Biol..

[bib62] Patergnani S., Bonora M., Ingusci S., Previati M., Marchi S., Zucchini S., Perrone M., Wieckowski M.R., Castellazzi M., Pugliatti M. (2021). Antipsychotic drugs counteract autophagy and mitophagy in multiple sclerosis. Proc. Natl. Acad. Sci. USA.

[bib63] Patergnani S., Castellazzi M., Bonora M., Marchi S., Casetta I., Pugliatti M., Giorgi C., Granieri E., Pinton P. (2018). Autophagy and mitophagy elements are increased in body fluids of multiple sclerosis-affected individuals. J. Neurol. Neurosurg. Psychiatr..

[bib92] Patergnani S., Marchi S., Rimessi A., Bonora M., Giorgi C., Mehta K.D., Pinton P. (2013). PRKCB/protein kinase C, beta and the mitochondrial axis as key regulators of autophagy. Autophagy.

[bib64] Patergnani S., Pinton P. (2015). Mitophagy and mitochondrial balance. Methods Mol. Biol..

[bib65] Peron C., Mauceri R., Cabassi T., Segnali A., Maresca A., Iannielli A., Rizzo A., Sciacca F.L., Broccoli V., Carelli V., Tiranti V. (2020). Generation of a human iPSC line, FINCBi001-A, carrying a homoplasmic m.G3460A mutation in MT-ND1 associated with Leber's Hereditary optic Neuropathy (LHON). Stem Cell Res..

[bib66] Quirós P.M., Mottis A., Auwerx J. (2016). Mitonuclear communication in homeostasis and stress. Nat. Rev. Mol. Cell Biol..

[bib67] Rabinovitch R.C., Samborska B., Faubert B., Ma E.H., Gravel S.P., Andrzejewski S., Raissi T.C., Pause A., St-Pierre J., Jones R.G. (2017). AMPK maintains cellular metabolic homeostasis through regulation of mitochondrial reactive oxygen species. Cell Rep..

[bib68] Ramos C.d.V.F., Bellusci C., Savini G., Carbonelli M., Berezovsky A., Tamaki C., Cinoto R., Sacai P.Y., Moraes-Filho M.N., Miura H.M.P.P. (2009). Association of optic disc size with development and prognosis of Leber's hereditary optic neuropathy. Invest. Ophthalmol. Vis. Sci..

[bib69] Sadun A.A., Win P.H., Ross-Cisneros F.N., Walker S.O., Carelli V. (2000). Leber's hereditary optic neuropathy differentially affects smaller axons in the optic nerve. Trans. Am. Ophthalmol. Soc..

[bib70] Scarpulla R.C. (2011). Metabolic control of mitochondrial biogenesis through the PGC-1 family regulatory network. Biochim. Biophys. Acta.

[bib71] Sharma L.K., Tiwari M., Rai N.K., Bai Y. (2019). Mitophagy activation repairs Leber's hereditary optic neuropathy-associated mitochondrial dysfunction and improves cell survival. Hum. Mol. Genet..

[bib72] Suski J.M., Lebiedzinska M., Bonora M., Pinton P., Duszynski J., Wieckowski M.R. (2012). Relation between mitochondrial membrane potential and ROS formation. Methods Mol. Biol..

[bib73] Thukral L., Sengupta D., Ramkumar A., Murthy D., Agrawal N., Gokhale R.S. (2015). The molecular mechanism underlying recruitment and insertion of lipid-anchored LC3 protein into membranes. Biophys. J..

[bib74] Toyama E.Q., Herzig S., Courchet J., Lewis T.L., Losón O.C., Hellberg K., Young N.P., Chen H., Polleux F., Chan D.C., Shaw R.J. (2016). Metabolism. AMP-activated protein kinase mediates mitochondrial fission in response to energy stress. Science.

[bib75] Tsujimoto Y., Shimizu S. (2005). Another way to die: autophagic programmed cell death. Cell Death Differ..

[bib76] Twig G., Shirihai O.S. (2011). The interplay between mitochondrial dynamics and mitophagy. Antioxid. Redox Signal..

[bib77] Vergani L., Martinuzzi A., Carelli V., Cortelli P., Montagna P., Schievano G., Carrozzo R., Angelini C., Lugaresi E. (1995). MtDNA mutations associated with Leber's hereditary optic neuropathy: studies on cytoplasmic hybrid (cybrid) cells. Biochem. Biophys. Res. Commun..

[bib78] Vives-Bauza C., Zhou C., Huang Y., Cui M., de Vries R.L.A., Kim J., May J., Tocilescu M.A., Liu W., Ko H.S. (2010). PINK1-dependent recruitment of Parkin to mitochondria in mitophagy. Proc. Natl. Acad. Sci. USA.

[bib79] Wallace D.C., Singh G., Lott M.T., Hodge J.A., Schurr T.G., Lezza A.M., Elsas L.J., Nikoskelainen E.K., Nikoskelainen E.K. (1988). Mitochondrial DNA mutation associated with Leber's hereditary optic neuropathy. Science.

[bib80] Wong A., Cortopassi G. (1997). mtDNA mutations confer cellular sensitivity to oxidant stress that is partially rescued by calcium depletion and cyclosporin A. Biochem. Biophys. Res. Commun..

[bib81] Wong R.C.B., Lim S.Y., Hung S.S.C., Jackson S., Khan S., Van Bergen N.J., De Smit E., Liang H.H., Kearns L.S., Clarke L. (2017). Mitochondrial replacement in an iPSC model of Leber's hereditary optic neuropathy. Aging.

[bib82] Wu W., Tian W., Hu Z., Chen G., Huang L., Li W., Zhang X., Xue P., Zhou C., Liu L. (2014). ULK1 translocates to mitochondria and phosphorylates FUNDC1 to regulate mitophagy. EMBO Rep..

[bib83] Xue J., Patergnani S., Giorgi C., Suarez J., Goto K., Bononi A., Tanji M., Novelli F., Pastorino S., Xu R. (2020). Asbestos induces mesothelial cell transformation via HMGB1-driven autophagy. Proc. Natl. Acad. Sci. USA.

[bib84] Yu-Wai-Man P., Griffiths P.G., Chinnery P.F. (2011). Mitochondrial optic neuropathies - disease mechanisms and therapeutic strategies. Prog. Retin. Eye Res..

[bib85] Yu-Wai-Man P., Griffiths P.G., Howell N., Turnbull D.M., Chinnery P.F. (2016). The epidemiology of leber hereditary optic neuropathy in the North East of England. Am. J. Hum. Genet..

[bib86] Yu-Wai-Man P., Soiferman D., Moore D.G., Burté F., Saada A. (2017). Evaluating the therapeutic potential of idebenone and related quinone analogues in Leber hereditary optic neuropathy. Mitochondrion.

[bib87] Yu J., Liang X., Ji Y., Ai C., Liu J., Zhu L., Nie Z., Jin X., Wang C., Zhang J. (2020). PRICKLE3 linked to ATPase biogenesis manifested Leber's hereditary optic neuropathy. J. Clin. Invest..

[bib88] Zaninello M., Palikaras K., Naon D., Iwata K., Herkenne S., Quintana-Cabrera R., Semenzato M., Grespi F., Ross-Cisneros F.N., Carelli V. (2020). Inhibition of autophagy curtails visual loss in a model of autosomal dominant optic atrophy. Nat. Commun..

[bib89] Zanna C., Ghelli A., Porcelli A.M., Martinuzzi A., Carelli V., Rugolo M. (2005). Caspase-independent death of Leber's hereditary optic neuropathy cybrids is driven by energetic failure and mediated by AIF and Endonuclease G. Apoptosis.

[bib90] Zmijewski J.W., Banerjee S., Bae H., Friggeri A., Lazarowski E.R., Abraham E. (2010). Exposure to hydrogen peroxide induces oxidation and activation of AMP-activated protein kinase. J. Biol. Chem..

[bib91] Lodi, R., Taylor, D.J., Tabrizi, S.J., Kumar, S., Sweeney, M., Wood, N.W., Styles, P., Radda, G.K., and Schapira, A.H. In vivo skeletal muscle mitochondrial function in Leber's hereditary optic neuropathy assessed by 31P magnetic resonance spectroscopy. Ann Neurol. 1997 Oct;42(4):573-9. doi: 10.1002/ana.410420407. PMID: 9382468. 10.1002/ana.410420407 9382468

