# Supplementary material for: Pathological mitophagy disrupts mitochondrial homeostasis in Leber’s hereditary optic neuropathy
Source: Cell Rep. 2022 Jul 19;40(3):111124. doi: 10.1016/j.celrep.2022.111124 (PMC9314546; doi:10.1016/j.celrep.2022.111124)
Supplement: Document S1. Figures S1–S6 and Tables S1 and S2 [file mmc1.pdf]

Cell Reports, Volume *40*

Supplemental information

Pathological mitophagy disrupts mitochondrial homeostasis in Leber's hereditary optic neuropathy

Alberto Danese, Simone Patergnani, Alessandra Maresca, Camille Peron, Andrea Raimondi, Leonardo Caporali, Saverio Marchi, Chiara La Morgia, Valentina Del Dotto, Claudia Zanna, Angelo Iannielli, Alice Segnali, Ivano Di Meo, Andrea Cavaliere, Magdalena Lebiedzinska-Arciszewska, Mariusz R. Wieckowski, Andrea Martinuzzi, Milton N. Moraes-Filho, Solange R. Salomao, Adriana Berezovsky, Rubens Belfort Jr., Christopher Buser, Fred N. Ross-Cisneros, Alfredo A. Sadun, Carlo Tacchetti, Vania Broccoli, Carlotta Giorgi, Valeria Tiranti, Valerio Carelli, and Paolo Pinton

Supplementary Material:

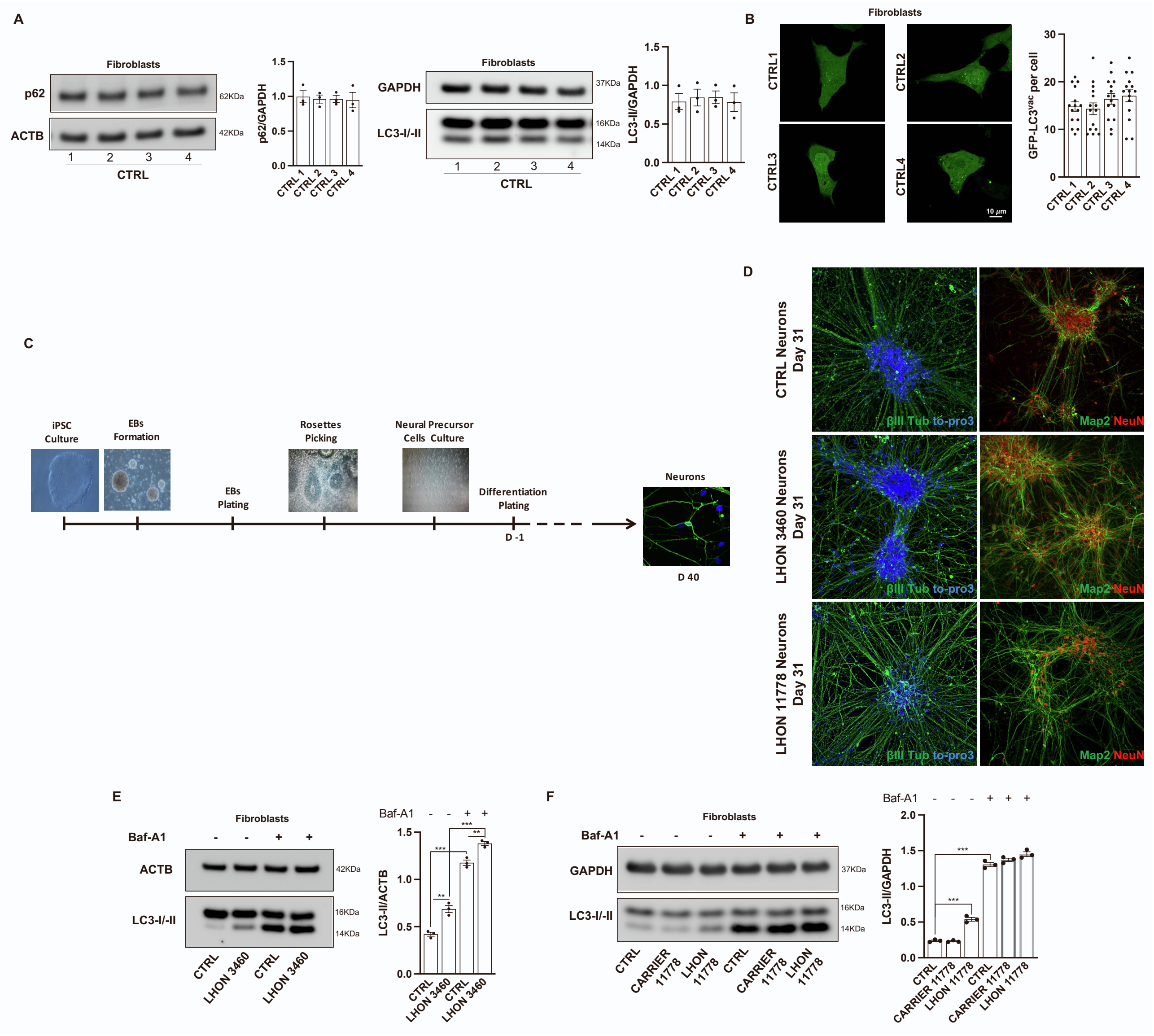

Figure S1: Comparative analysis of autophagy levels in all control fibroblasts used for this study (**A-B**). Neural differentiation timeline, adapted from Brafman et al. 2014 (**C**). Control and patients' neurons characterization by immunofluorescence, showing specific markers like βIII-Tub, MAP2 and NEUN positive expression (**D**). Autophagy flux analysis using a Bafilomycin A1 (Baf-A1) treatment in complete medium for 2 hours in fibroblasts harboring 3460 and 11778 mutations (**E-F**). Data are presented as means ± SEM. n = at least 3 independent experiments for Western Blots or 5 visual fields per at least 3 independent samples per condition for fluorescent microscopy experiments. * $p<0.05$, ** $p<0.01$ and *** $p<0.001$.

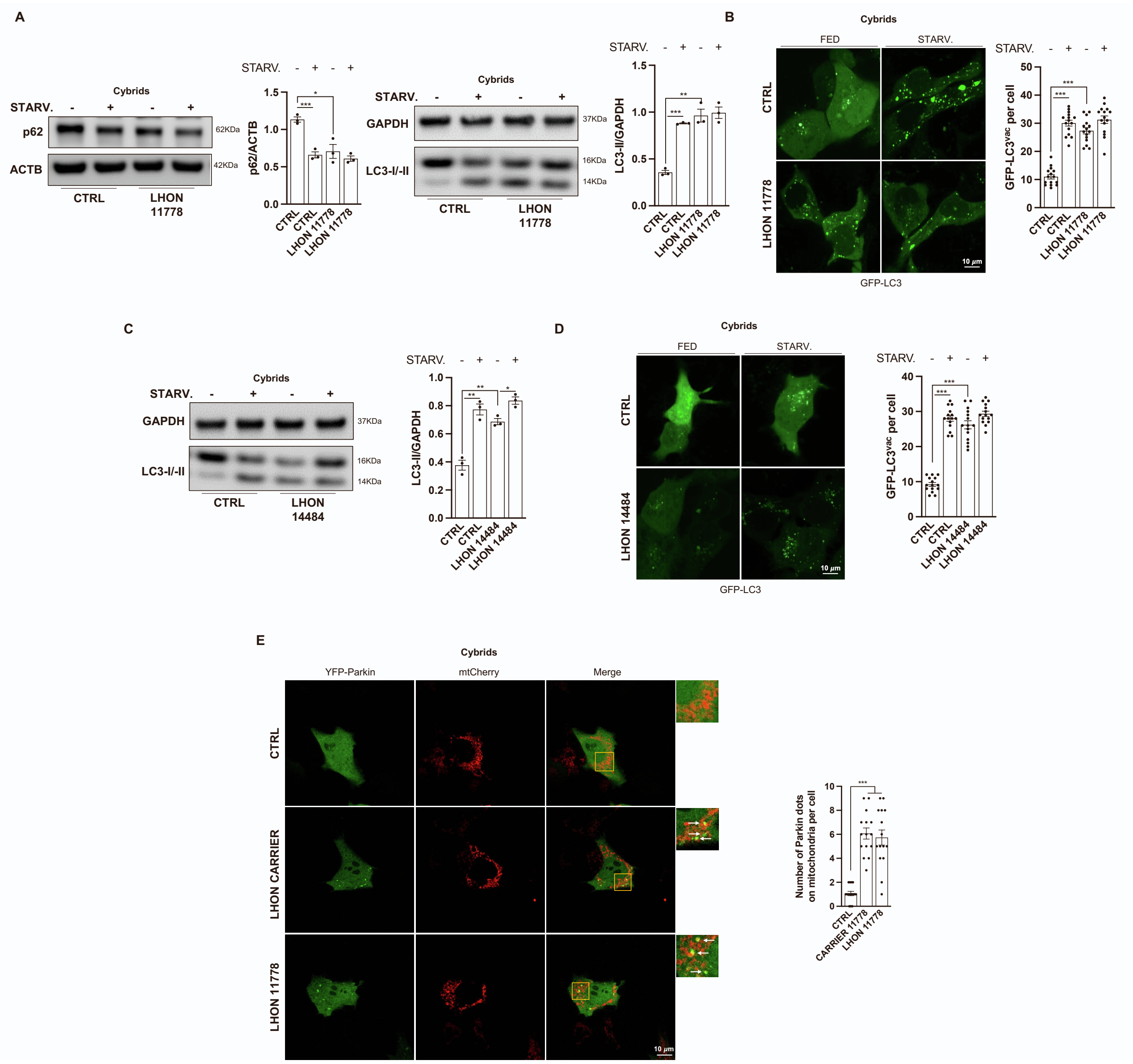

Figure S2: Detection of autophagy activity through immunoblot and GFP-LC3 puncta count on a second 11778 LHON cybrid line (**A-B**) and in LHON cybrids carrying 14484 mutation (**C-D**). Mitophagy levels are assessed by detecting the amount of fluorescent mitochondria-localized YFP-Parkin in LHON and Carrier fibroblasts-derived cybrids (**E**). Data are presented as means ± SEM. n = at least 3 independent experiments for Western Blots or 5 visual fields per at least 3 independent samples per condition for fluorescent microscopy experiments. * $p<0.05$, ** $p<0.01$ and *** $p<0.001$.

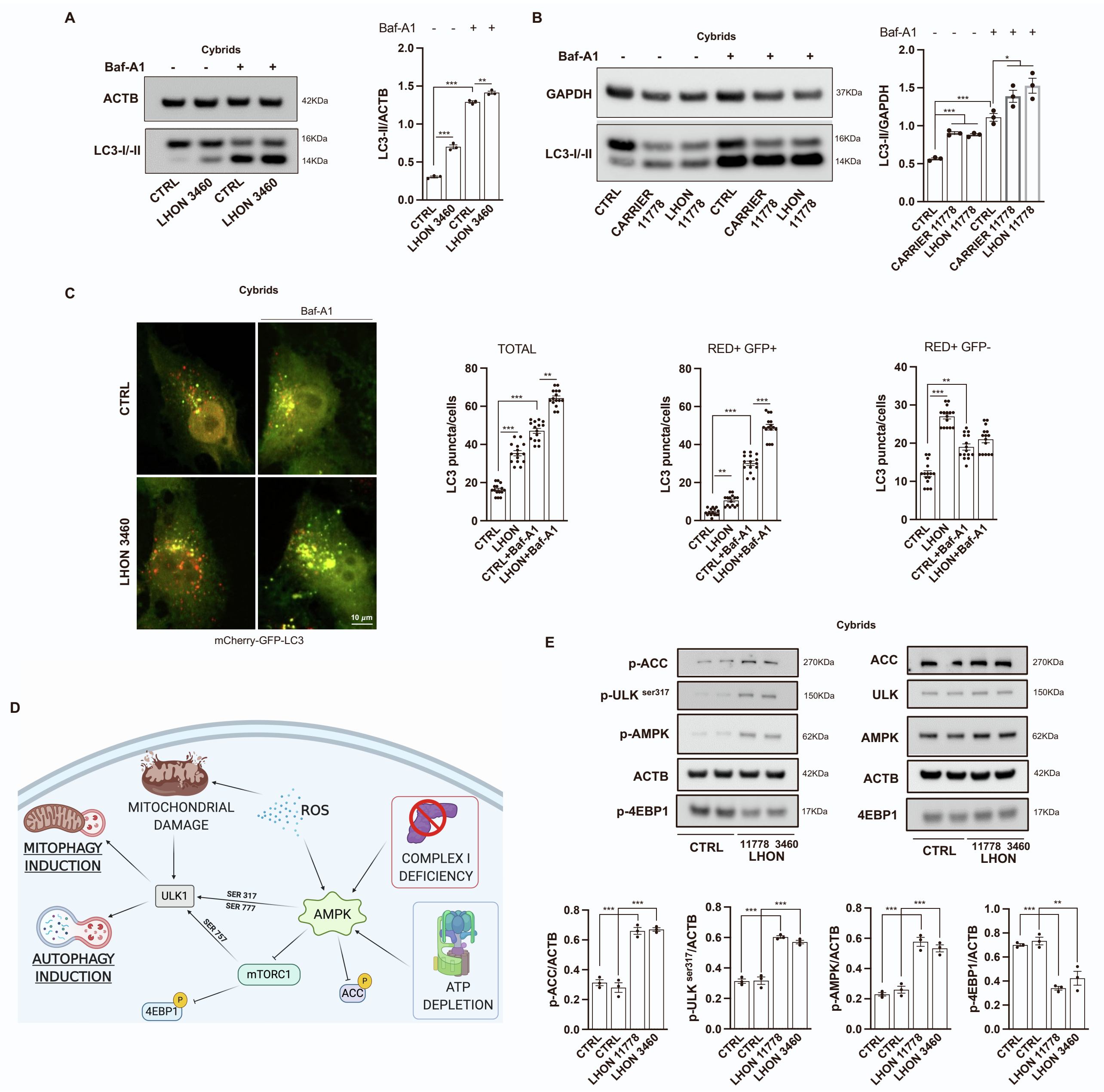

Figure S3: Autophagy flux analysis using a Bafilomycin A1 (Baf-A1) treatment in complete medium for 2 hours in cybrids harboring 3460 (**A**) and 11778 (**B**) mutations. Autophagy flux was also assessed by expressing mCherry-GFP-LC3 construct in LHON cybrids (**C**). Representative scheme of the proteins involved in the mTOR/AMPK pathway (**D**) that was assessed by analyzing the expression of its components (**E**). Data are presented as means ± SEM. n = at least 3 independent experiments for Western Blots or 5 visual fields per at least 3 independent samples per condition for fluorescent microscopy experiments. * $p<0.05$, ** $p<0.01$ and *** $p<0.001$.

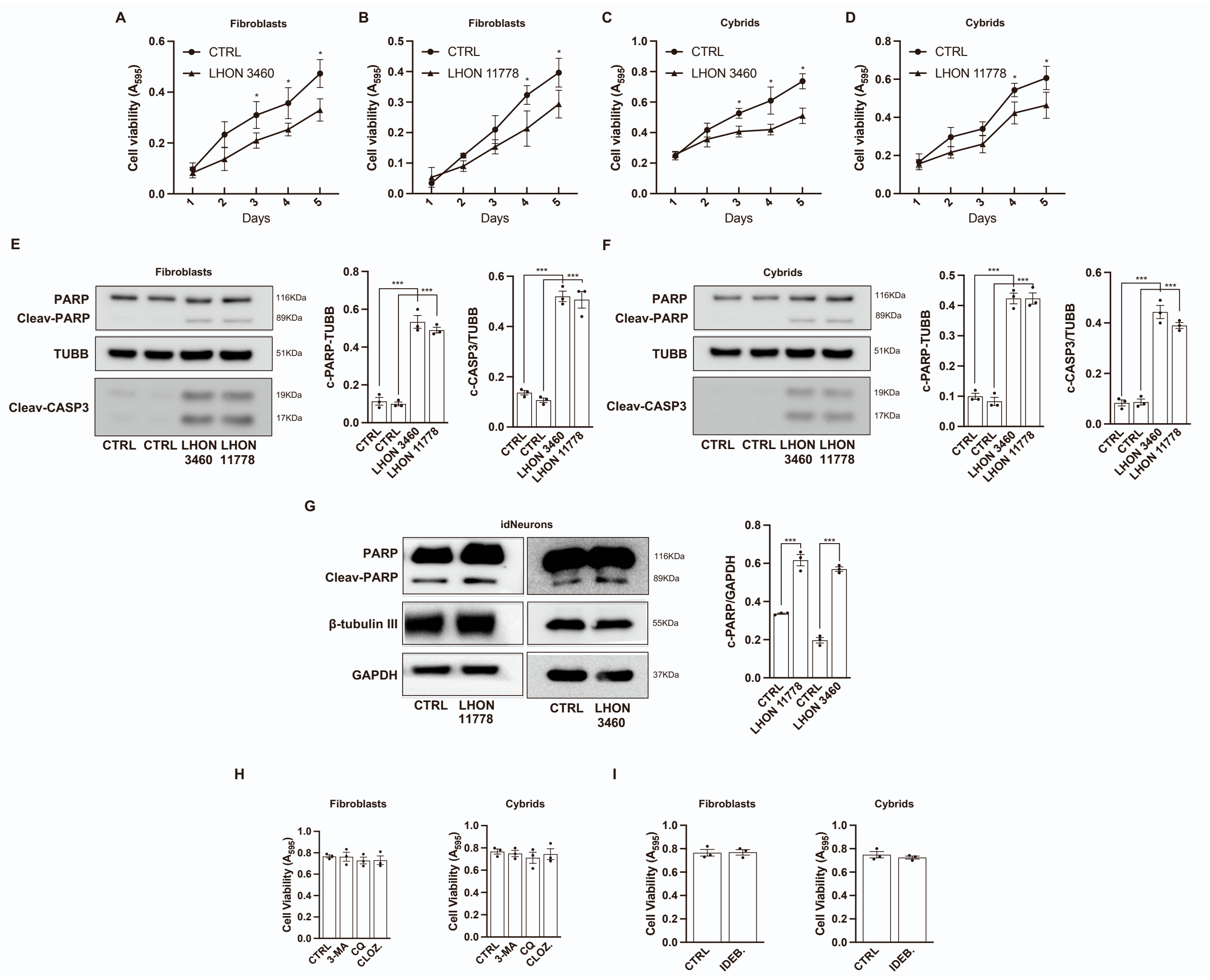

Figure S4: **LHON mutations give a higher predisposition to apoptotic death.** Cell proliferation growth curves performed in fibroblasts (**A-B**) and cybrids (**C-D**) carrying 3460 and 11778 mutations, respectively. Detection of apoptotic process by immunoblotting with antibodies against PARP and CAS3 apoptotic markers in fibroblasts (**E**) and cybrids (**F**) with 3460 and 11778 mutations. Immunoblot with antibodies against the apoptotic marker PARP to assess apoptotic activity on idNeurons with 3460 and 11778 mutations (**G**). Cell viability assay performed in control fibroblasts and cybrids pretreated with anti-autophagic compounds (**H**) and Idebenone (**I**). Treatments were carried out according to times and concentrations described in materials and methods section.

Data are presented as means ± SEM. n = at least 3 independent experiments. * $p<0.05$; ** $p<0.01$; *** $p<0.001$.

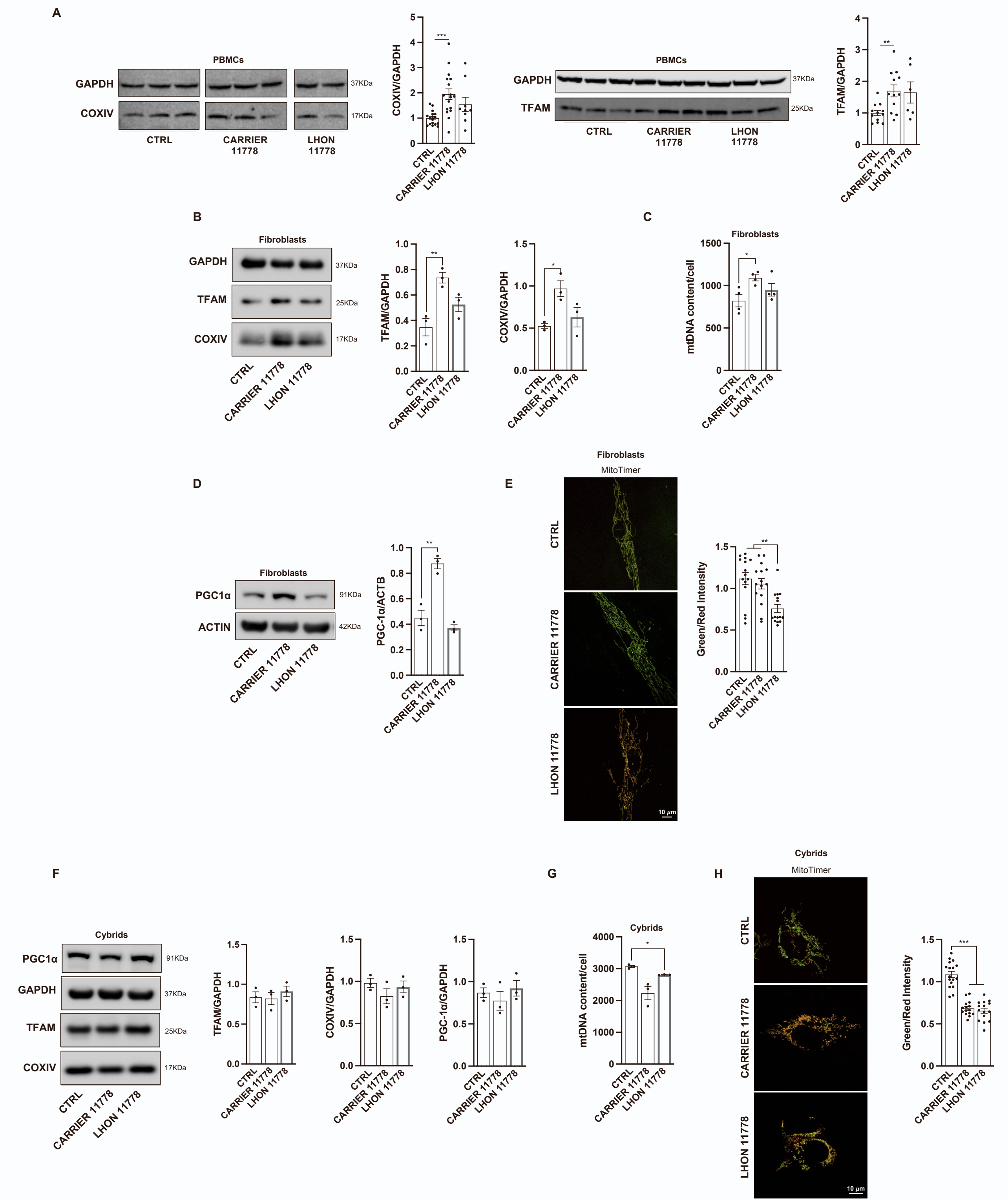

Figure S5: Mitochondrial biogenesis evaluated in *ex-vivo* PBMCs by immunoblot of COX-IV and TFAM proteins. For COX-IV assessment, 17 healthy individuals (CTRLs), 17 LHON 11778-carrier and 9 LHON 11778- affected patients were analyzed; for TFAM assessment, 12 CTRLs, 12 LHON

11778-carrier and 6 LHON 11778-affected patients were analyzed Representative images and densitometric analysis are shown (the representative image shown in the left panel has been cropped to invert the order of samples loading) (**A**) Mitochondrial mass was also measured in fibroblasts by immunoblot (**B**) and by detecting the mtDNA abundance (**C**). Representative immunoblot showing the different pattern of protein expression of PGC1-α in CTRL, LHON-carrier and LHON-affected fibroblasts (**D**). The mitochondrial turnover was investigated by using the fluorescent plasmid MitoTimer. The graph depicts the ratio between the green and red intensity, respectively indicative for newly synthetized and old mitochondria (**E**). Mitochondrial mass and PGC1-α expression in cybrids generated starting from fibroblasts derived from one LHON affected patient carrying the 11778 mutation and of the non-affected (carrier) brother carrying the same 11778 mutation was analyzed by immunoblot (**F**) and by detecting the mtDNA levels (**G**). The same cells were used to analyze the mitochondrial turnover rates by using the fluorescent plasmid MitoTimer (**H**). Data are presented as means ± SEM. n = at least 3 independent experiments for Western Blots or 5 visual fields per at least 3 independent samples per condition for fluorescent microscopy experiments. * $p<0.05$, ** $p<0.01$ and *** $p<0.001$.

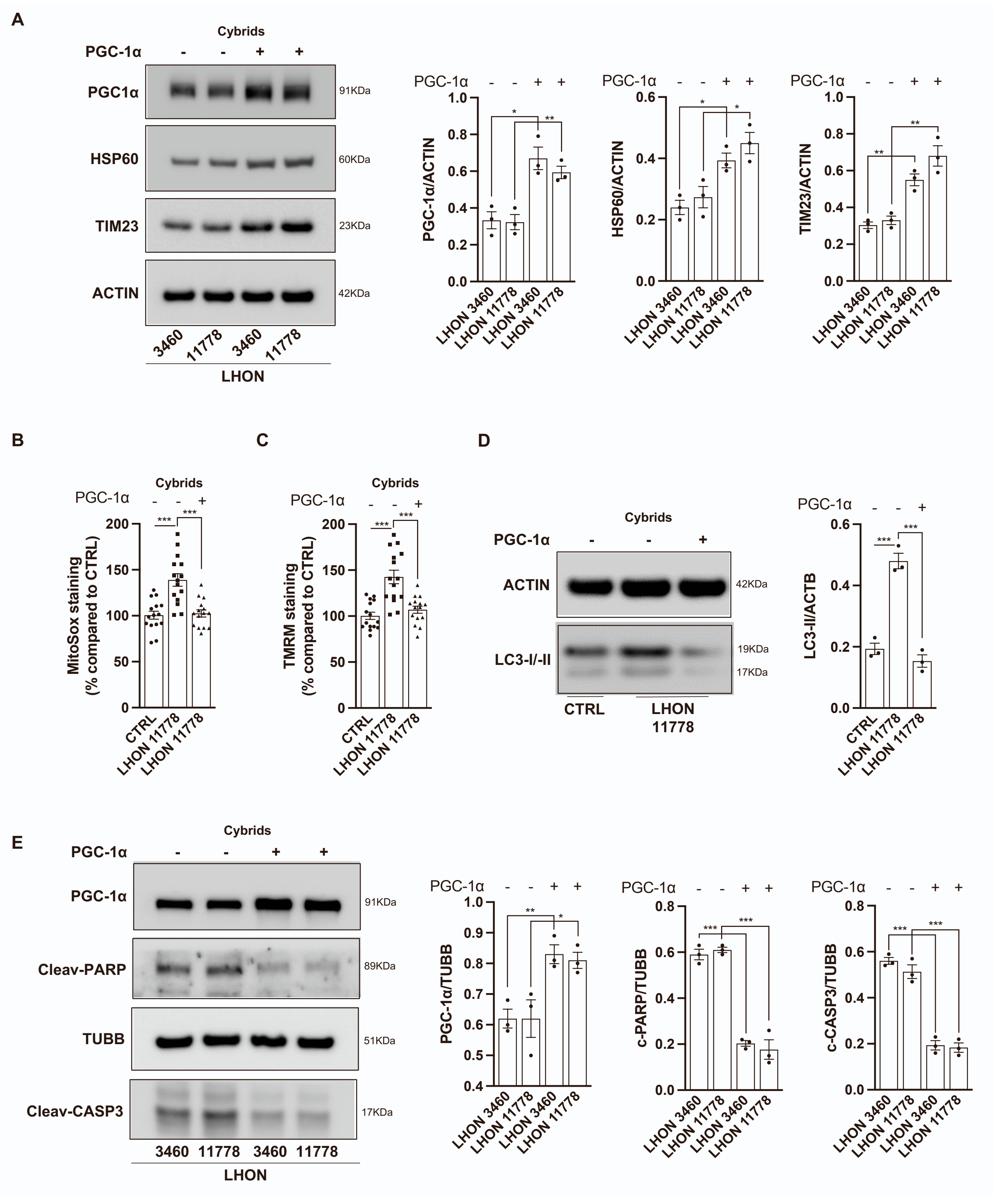

Figure S6: The effect of PGC1-α overexpression in mitochondrial biogenesis in 3460 and 11778 cybrids was confirmed by immunoblot (**A**). Measurements of mitochondrial ROS production (**B**) and mitochondrial transmembrane potential (**C**) were studied in 11778 cybrids expressing PGC1-α. PGC1-α not only stimulates mitochondrial functioning, but also reduces the excessive autophagy

levels (**D**) and apoptotic features (**E**) of LHON cybrids. Data are presented as means ± SEM. n = at least 3 independent experiments for Western Blots or 5 visual fields per at least 3 independent samples per condition for fluorescent microscopy experiments. * $p<0.05$, ** $p<0.01$ and *** $p<0.001$.

Supplementary Table 1: mtDNA complete sequence of cell lines. Related to STAR Methods.

	Cell lines	mtDNA mutation	Haplotype[1]	Private variants
**Cybrids**	HPS11	CTRL	T2	m.152T>C/MT-HV2<br>m.549C>T/MT-HV2<br>m.5438C>T/MT-ND2, syn<br>m.7471del/MT-TS1<br>m.10750C>T/MT-ND4L, p.N94A
	HGA2	CTRL	J1c7a	m.146T>C/MT-HV2
	HL180	14484	J1c2j2	m.14279G>A/MT-ND6, p.S132L<br>**m.14484T>C/MT-ND6, p.M64L**
	RJ206	3460	T1a1	**m.3460G>A/MT-ND1, p.A52T**
	HPE9	11778	J1c4	**m.11778G>A/MT-ND4, p.R340H**<br>m.11914G >A/MT-ND4, syn
	C45L	11778	T2b	m.8084A>G/MT-CO2, p.T167A<br>m.8794C>T/MT-ATP6, p.H90Y<br>**m.11778G>A/MT-ND4, p.R340H**<br>m.15446C>T/MT-CYB, p.L234F<br>m.16240A>C/MT-HV1
	C46L	Carrier 11778	T2b	m.8084A>G/MT-CO2, p.T167A<br>m.8794C>T/MT-ATP6, p.H90Y<br>**m.11778G>A/MT-ND4, p.R340H**<br>m.15446C>T/MT-CYB, p.L234F<br>m.16240A>C/MT-HV1
**Fibroblasts**	ZAMAC	CTRL	T2d1b2	m.195T>C/MT-HV2
	PAVE	CTRL	H1	m.5075T>C/MT-ND2, syn<br>m.6815T>C/MT-CO1, syn<br>m.10377C>T/MT-ND3, syn<br>m.15618T>C/MT-CYB, p.V291A<br>m.16093T>C/MT-HV1
	F15W	CTRL	H	m.146T>C/MT-HV2<br>m.3531G>A/MT-ND1, syn<br>m.16093T>C/MT-HV1<br>m.16311T>C/MT-HV1
	F08W	CTRL	T2b5	m.151C>T/MT-HV2<br>m.9948G>A/MT-CP3, p.V248I
	F45L	11778	T2b	m.8084A>G/MT-CO2, p.T167A<br>m.8794C>T/MT-ATP6, p.H90Y<br>**m.11778G>A/MT-ND4, p.R340H**<br>m.15446C>T/MT-CYB, p.L234F<br>m.16240A>C/MT-HV1
	F46L	Carrier 11778	T2b	m.8084A>G/MT-CO2, p.T167A<br>m.8794C>T/MT-ATP6, p.H90Y<br>**m.11778G>A/MT-ND4, p.R340H**<br>m.15446C>T/MT-CYB, p.L234F<br>m.16240A>C/MT-HV1
	F33L	11778	H82	m.152T>C/MT-HV2<br>m.195T>C/MT-HV2<br>m.3397A>G/MT-ND1, p.M31V<br>**m.11778G>A/MT-ND4, p.R340H**<br>m.16176C>T /MT-HV1
	F34L	Carrier 11778	H82	m.152T>C/MT-HV2<br>m.195T>C/MT-HV2<br>m.3397A>G/MT-ND1, p.M31V<br>**m.11778G>A/MT-ND4, p.R340H**<br>m.16176C>T /MT-HV1

**Fibroblasts**	F56L	3460	H1at1	**m.3460G>A/MT-ND1, p.A52T**<br>m.6176T>C/MT-CO1, syn<br>m.8863G>A/MT-ATP6, p.V113M<br>m.9311T>C/MT-CO3, syn<br>m.12460T>C/MT-ND5, p.S42A h42%

Note: [1] Haplotype according to PhyloTree.org - mtDNA tree Build 17 (18 Feb 2016)

The mtDNA reference sequence used for variant calling was the rCRS (NC_012920.1). For each cell lines, cybrids and fibroblasts, the haplotype were assigned according to PhyloTree.org - mtDNA tree Build 17 (18 Feb 2016) and the variant not marker of haplotype were defined as private variants.

Supplementary Table 2: Table collecting the p-values of histograms that did not reach statistical significance in any of the conditions examined. Related to Figure 1, S1, S5 and S6.

Figure	Conditions	p-value
**Figure 1H**	CTRL vs CARRIER 11778	0.38
	CTRL vs LHON 11778	0.18
	CARRIER 11778 vs LHON 11778	0.51
**Figure S1A (p62)**	CTRL1 vs CTRL2	0.75
	CTRL1 vs CTRL 3	0.75
	CTRL1 vs CTRL 4	0.74
	CTRL2 vs CTRL3	0.99
	CTRL2 vs CTRL4	0.91
	CTRL3 vs CTRL4	0.91
**Figure S1A (LC3-II)**	CTRL1 vs CTRL2	0.74
	CTRL1 vs CTRL 3	0.69
	CTRL1 vs CTRL 4	0.97
	CTRL2 vs CTRL3	0.98
	CTRL2 vs CTRL4	0.73
	CTRL3 vs CTRL4	0.69
**Figure S1B**	CTRL1 vs CTRL2	0.82
	CTRL1 vs CTRL 3	0.45
	CTRL1 vs CTRL 4	0.37
	CTRL2 vs CTRL3	0.38
	CTRL2 vs CTRL4	0.31
	CTRL3 vs CTRL4	0.84
**Figure S5H (Fibroblasts)**	CTRL vs 3MA	0.95
	CTRL vs CQ	0.34
	CTRL vs CLOZ.	0.45
	3MA vs CQ	0.54
	3MA vs CLOZ.	0.6
	CQ vs CLOZ.	0.97
**Figure S5H (Cybrids)**	CTRL vs 3MA	0.65
	CTRL vs CQ	0.37
	CTRL vs CLOZ.	0.69
	3MA vs CQ	0.54
	3MA vs CLOZ.	0.93
	CQ vs CLOZ.	0.66
**Figure S5I (Fibroblasts)**	CTRL vs IDEB.	0.92
**Figure S5I (Cybrids)**	CTRL vs IDEB.	0.47
**Figure S6F (TFAM)**	CTRL vs CARRIER 11778	0.88
	CTRL vs LHON 11778	0.49
	CARRIER 11778 vs LHON 11778	0.42
**Figure S6F (COXIV)**	CTRL vs CARRIER 11778	0.2
	CTRL vs LHON 11778	0.62
	CARRIER 11778 vs LHON 11778	0.38
**Figure S6F (PGC-1α)**	CTRL vs CARRIER 11778	0.49
	CTRL vs LHON 11778	0.66
	CARRIER 11778 vs LHON 11778	0.36
